# Identification of mental disorders in South Africa using complex probabilistic hesitant fuzzy N-soft aggregation information

**DOI:** 10.1038/s41598-023-45991-7

**Published:** 2023-11-16

**Authors:** Shahzaib Ashraf, Muneeba Kousar, Gilbert Chambashi

**Affiliations:** 1 Institute of Mathematics, Khwaja Fareed University of Engineering & Information Technology, Rahim Yar Khan, 64200 Pakistan; 2 School of Business Studies, Unicaf University, Longacres, Lusaka Zambia

**Keywords:** Health care, Mathematics and computing

## Abstract

This paper aims to address the challenges faced by medical professionals in identifying mental disorders. These mental health issues are an increasing public health concern, and middle-income nations like South Africa are negatively impacted. Mental health issues pose a substantial public health concern in South Africa, putting forth extensive impacts on both individuals and society broadly. Insufficient funding for mental health remains the greatest barrier in this country. In order to meet the diverse and complex requirements of patients effective decision making in the treatment of mental disorders is crucial. For this purpose, we introduced the novel concept of the complex probabilistic hesitant fuzzy N-soft set (CPHFNSS) for modeling the unpredictability and uncertainty effectively. Our approach improves the precision with which certain traits connected to different types of mental conditions are recognized by using the competence of experts. We developed the fundamental operations (like extended and restricted intersection, extended and restricted union, weak, top, and bottom weak complements) with examples. We also developed the aggregation operators and their many features, along with their proofs and theorems, for CPHFNSS. By implementing these operators in the aggregation process, one could choose a combination of characteristics. Further, we introduced the novel score function, which is used to determine the optimal choice among them. In addition, we created an algorithm with numerical illustrations for decision making in which physicians employ CPHFNS data to diagnose a specific condition. Finally, comparative analyses confirm the practicability and efficacy of the technique that arises from the model developed in this paper.

## Introduction

Decision-making is a common challenge for many types of businesses, including manufacturing, the armed forces, finance, the medical field, and others. Choosing among a set of possibilities according to a set of criteria is one example of such a challenge. If alternative options are based only on criteria, the decision-making process is fairly simple. If there are several criteria for which each possibility has a distinct value, however, an aggregating technique is required to get a single value for each option. Multi-parameter Group Decision Making (MPGDM) will address this difficulty in the decision-making system. The increasing complexity of decision-making is attributed to the significant level of ambiguity inherent in information, especially when it is presented in a yes-or-no kinds as opposed to more or fewer types. In conventional logic, a proposition may only be true or false; there is no in-between.

Zadeh^1^ developed the fuzzy theory to circumvent these restrictions. It is meant to symbolize the deterministic uncertainty for which he created the idea of the membership function. Decision-making is considerably facilitated by fuzzy theories. But nonetheless, individuals sometimes struggled to handle group decision-making challenges when they were required to pick among several potential membership grades for an element in the group. Consider the case below: Two experts are disputing whether x falls in category A, with one wanting to offer a score of 0.6 and the other desiring to offer a score of 0.7. As a consequence, there is a degree of ambiguity around the range of probable values. Torra^2^ designed a hesitant fuzzy set to cope with uncertain circumstances in order to circumvent these constraints. In this instance, he constructed the membership function as a collection of potential membership values, h(x), where h(x) is a discrete subset of [0, 1]. With the passage of time, hesitant fuzzy sets have gained popularity and been used for a number of tasks, such as clinical diagnosis, retrieval of information, clustering, and decision-making concerns that play a significant role. Researchers introduced the dual-hesitant fuzzy soft set model for group forecasting. They utilized the pharmaceutical firm as an example, in which the board had to set the priorities for future investments in subsidiaries based on net income^3^, a wind power project site selection algorithm based on hesitancy^4,5^, and decision-making^6,7^ etc.

In many real-world circumstances, however, individuals encounter a mix of deterministic and random factors while making judgments. For example, weather patterns are both somewhat unpredictable and partially random. Meghdadi^8^ established the notion of probabilistic fuzzy logic to address these types of scenarios. In applications including robotic control systems^9^, signal processing^10^, fog-haze factor assessment problems^11^, power systems^12^, control systems^13^, drug selection to treat COVID-19^14^, and decision-making^15–17^. Probabilistic modelling is an essential instrument for managing random uncertainty. Several scientists study probabilistic fuzzy set hybrid models such as^18–20^, which play a crucial role in resolving real-world issues. It has been discovered from the aforementioned ideas that the fuzzy set and its generalizations are only capable of addressing vagueness and ambiguity in data when analyzing decision-making concerns. They cannot accurately show the variances in data at a particular time period. Ramot^21^ suggested a complex fuzzy set to address these limitations. Instead of being limited to the range [0, 1], this range extends to the complex plane’s unit circle, unlike a standard fuzzy membership function. The complex fuzzy set caught the attention of scholars, who subsequently created several hypotheses in relation to it. For instance, its operation and equalities^22^, distance measures of complex Pythagorean fuzzy sets and their applications in pattern recognition^23^, complex fuzzy computing for time series prediction^24^, for simulator selection^25,26^ and many more.

According to Molodtsov, a significant difficulty with these theories is their poor use of parameterization tools, such as the individual’s capacity to evaluate their degree of membership depending on the information they receive, which makes them prone to subjective viewpoints. In addition, it is essential to analyze all aspects of a situation concurrently. To circumvent these limits, Molodtsov developed a soft-set computing method^27^. The soft set theory provides the benefit of having problems that take several factors into consideration. It offers tremendous potential for problem-solving and is crucial to a wide range of sectors^28,29^. Researchers improved and expanded soft set theory. Babitha and Sunil explained the look of a fuzzy soft set and its relationships and functions^30^. Many researchers used soft set theory for decision-making problems^31–33^ rule mining^34^, energy^35^, and business^36^. Rating or ranking systems frequently use non-binary evaluations. Real-world examples demonstrate how ratings may take the form of a star rating, such as “five stars”, “four stars”, “three stars”, “two stars”, or “one star”. For this, Fatimah et al.^37^ expanded the soft set model and presented the idea of the N-soft set and discussed the great importance of ordered grades in practical situations. They created decision-making processes for the N-soft set as well. The semantics of N-soft sets are now well studied with the help of the article^38^. The work on N-soft set theory by Akram et al.^39^ is remarkable. The theory of N-soft sets plays an interesting and advantageous role; therefore, many scholars work with this model, such as hesitant fuzzy N-soft sets^40^, complex fermatean fuzzy N-soft sets^41^, reduction of N-soft sets^42^, complex pythagorean fuzzy N-soft sets^43^ and belief interval-valued N-soft sets^44^ and complex spherical fuzzy N-soft sets^45^.

In the vast majority of African countries, mental illness is considered a “silent catastrophe”. Mental illness has been described as a neglected and growingly onerous issue impacting all sectors of Africa’s population^46,47^. It has also been challenging to prioritize mental health owing to a lack of resources, restricted financing, and inadequate or nonexistent mental health policies^48^. Across the African Region, more than 116 million people were already estimated to be living with mental health conditions pre-pandemic, and pandemic-19 places a double load on the country’s already frail healthcare system^49^. Mental disorders are prevalent in South Africa, with an estimated 30% of the population experiencing a mental disorder at some point in their lives. This high prevalence highlights the need for effective identification and treatment of mental disorders in the country. These have a significant impact on society, including decreased productivity, increased healthcare costs, and reduced quality of life^50^. Identifying mental disorders and providing appropriate treatment can help mitigate these negative effects and improve the overall well-being of individuals and society as a whole. Hence, a system that can accommodate doctors’ diagnostic preferences is essential. The secondary purpose of this article is to widen the application of the N-soft set theory, in which grades produce a parameterized interpretation of alternatives to the universe. This is important because individuals sometimes have trouble making decisions when confronted with the fuzziness, hesitation, and unpredictability of uncertain evidence combined with a parameterized family of subsets and grades of alternatives in the universe. For example, physicians confront several challenges while treating mental disorders. A patient with a mental condition is often treated by many physicians, which creates fuzziness, hesitancy, and randomness and makes diagnosis difficult. Consider a 30-year-old man/woman statement, who seeks bipolar disorder therapy at a multi-physician mental health facility. His/Her symptoms change over time, making diagnosis difficult. Some doctors think he/she has bipolar illness, but others don’t since her symptoms are unusual, such as combined spells of mania and despair, introducing fuzziness into the diagnosis. Treatment decisions exhibit hesitancy, as some physicians recommend mood stabilizers, while others recommend a combination of medicine and psychotherapy, indicating doubt regarding the best treatment, and reflecting uncertainty about the most effective approach. His/Her response to the therapy exhibits an element of unpredictability, characterized by intermittent phases of stability that are afterwards interrupted by unintentional relapses. This unpredictability introduces a degree of randomness to the management of her condition.

In this study, a model for healthcare group decision-making has been developed. Using the model of a complex probabilistic hesitant fuzzy N-soft set, medical professionals can acquire patient data in a more effective manner. In which the amplitude term reflects the degree to which a symptom is associated with the illness in question, the phase term is generally connected to the periodicity or length of the appearance of the disease or symptoms, the grading indicates the severity, and the probability shows the likelihood that the disease or symptoms will really manifest. However, hesitant fuzzy approximations enhance the flexibility and depth of the decision-analysis process. We also described the technique or algorithm used to process this data so that physicians get a list of disorders from worst to least, which reflects the disease hierarchy. The suggested approach provides a variety of solutions based on motivation and the addressed issue. The potential advantages of the proposed concepts are:

1. *Improved accuracy* Mental disorders are complex and multifaceted, making their identification a challenging task. CPHFNSS can provide a more accurate representation of the uncertainty and imprecision inherent in mental disorder diagnosis.
2. *Improved efficiency* Mental disorders can manifest in different ways and have varying degrees of severity. This model leads to more efficient algorithms or decision-making processes by reducing the complexity of the problem or increasing the speed of computation and allows a flexible and adaptable approach to diagnosis, which can account for these variations in symptoms and severity.
3. *Improved interpretability* This model provides a clear and interpretable representation of the diagnostic process, which can help physicians to understand and communicate the reasoning behind their diagnosis. This leads to better collaboration between physicians and improved patient outcomes.
4. *Incorporation of hesitancy* Mental disorder diagnosis is often characterized by uncertainty and hesitancy, with physicians being uncertain about the presence or absence of a particular symptom. This model allows physicians to express this hesitancy in a systematic and structured way, which can improve the diagnostic process.

Clearly, the modelization provided by present theories is inadequate to account for these scenarios as represented in Table 11. To achieve the aforementioned goals, the remaining sections of this work is organized as follows.

Preliminaries are discussed in “Preliminaries” section, where we carefully review a few fundamental definitions and operations to help us explain more easily the following sections. The notion of a complex probabilistic hesitant fuzzy N-soft set is developed in “Complex probabilistic hesitant fuzzy N-soft set” section. We talked about novel score function and various fundamental operations, including extended and restricted intersection, extended and restricted union, weak complement, top and bottom weak complement, as well as aggregation operations. Additionally, confirm their essential laws. Furthermore, the numerical examples have been resolved to demonstrate the validity and superiority of the research work. In “Aggregation operators for CPHFNSS” section, we developed the averaging and geometric aggregations operators, its theorems and properties. In “Framework of multi-parameter group decision making” section, we developed the decision-making algorithm based on complex probabilistic hesitant fuzzy N-soft information and also illustrate the numerical example to help readers make the best choices. In “Comparison analysis” section we discussed the comparative analysis with the existing studies. While we provided the paper’s conclusion in “Conclusion” section.

## Preliminaries

The definitions we used to establish the methods in this paper are briefly reviewed in this section.

### Definition 2.1

If $\mathbb {Z}$ be a universal set then ${{\mathbb {A}}}$ is a fuzzy set defined on $\mathbb {Z}$ as:

$$\begin{aligned} {{\mathbb {A}}} = \Big \{\langle \rho _{i},\mu _{{{\mathbb {A}}}}(\rho _{i})\rangle \big | \rho _{i}\in \mathbb {Z} \Big \}, \end{aligned}$$

where $\mu _{{{\mathbb {A}}}}(\rho _{i})$ is a membership degree of $\rho _{i}$ in ${{\mathbb {A}}}$ and $\mu _{{{\mathbb {A}}}} : \mathbb {Z} \rightarrow [0, 1]$. If $\mu _{{{\mathbb {A}}}}(\rho _{i}) = 0$, is considered to be non-membership of $\rho _{i}$. If $\mu _{{{\mathbb {A}}}}(\rho _{i}) = 1$, is considered to be the entire membership of $\rho _{i}$. If $\mu _{{{\mathbb {A}}}}(\rho _{i})$ value among 0 and 1, is considered to be partial membership of $\rho _{i}$^1^.

### Definition 2.2

If $\mathbb {Z}$ be a universal set then ${{\mathbb {B}}}$ is a hesitant fuzzy set in terms of the *h* function, which returns a subset of [0,1] when it applies to $\mathbb {Z}$ which is denoted as:

$$\begin{aligned} {{\mathbb {B}}} = \Bigg \{\bigg \langle \rho _{i},h_{{{\mathbb {B}}}}(\rho _{i})\bigg \rangle \big | \rho _{i}\in \mathbb {Z} \Bigg \}, \end{aligned}$$

where $h_{{{\mathbb {B}}}}(\rho _{i})$ is the collection of distinct finite elements in [0,1], represents the possible membership degrees of the element $\rho _{i}\in \mathbb {Z}$ to the set ${{\mathbb {B}}}$^2^.

### Definition 2.3

If $\mathbb {Z}$ be a universal set then ${{\mathbb {C}}}$ is a probabilistic hesitant fuzzy set defined as:

$$\begin{aligned} {{\mathbb {C}}} = \Bigg \{\bigg \langle \rho _{i},h_{{{\mathbb {C}}}}\Big (\mu _{m} (\rho _{i})\big |P_{{{\mathbb {C}}}_{m}}\Big )\bigg \rangle \big | \rho _{i}\in \mathbb {Z} \Bigg \}, \end{aligned}$$

where $h_{ {{\mathbb {C}}}}\big (\mu _{m} (\rho _{i})|P_{{{\mathbb {C}}}_{m}}\big )$ is the collection of distinct finite elements $(\mu _{m} (\rho _{i})\big |P_{{{\mathbb {C}}}_{m}})$ representing the hesitant fuzzy information along probabilities to the set ${{\mathbb {C}}}, m = 1, 2, 3,...,l$ where *l* is the number of possible elements in $h_{ {C}}\big (\mu _{m} (\rho _{i})|P_{{{\mathbb {C}}}_{m}}\big ), P_{{{\mathbb {C}}}_{m}}\in [0,1]$ is the hesitant probability of $\rho _{i}$ and $\sum \limits _{m} P_{{{\mathbb {C}}}_{m}}= 1$^51^.

### Definition 2.4

If $\mathbb {Z}$ be a universal set then ${{\mathbb {D}}}$ is a complex fuzzy set defined as:

$$\begin{aligned} {{\mathbb {D}}} = \Bigg \{\bigg \langle \rho _{i},r_{{{\mathbb {D}}}}(\rho _{i})e^{2\pi i \psi _{{{\mathbb {D}}}}(\rho _{i})}\bigg \rangle \big | \rho _{i}\in \mathbb {Z} \Bigg \}, \end{aligned}$$

where $r_{{{\mathbb {D}}}}(\rho _{i})e^{2\pi i \psi _{{{\mathbb {D}}}}(\rho _{i})}$ is a complex valued membership degree of $\rho _{i}$ in ${{\mathbb {D}}}$ and it might be given any value that is contained in the complex plane unit circle. and $r_{{{\mathbb {D}}}}(\rho _{i})\in [0,1]$, $\psi _{{{\mathbb {D}}}}(\rho _{i}) \in [0,1]$ and $i = \sqrt{-1}$^21^.

### Definition 2.5

If $\mathbb {Z}$ be a universal set then ${{\mathbb {E}}}$ is a complex probabilistic hesitant fuzzy set (CPHFS) defined as:

$$\begin{aligned} {{\mathbb {E}}} = \Bigg \{\bigg \langle \rho _{i},h_{{{\mathbb {E}}}}\bigg (r_{{{\mathbb {E}}}_{m}}(\rho _{i})e^{2\pi i \psi _{{{\mathbb {E}}}_{m}}(\rho _{i})}\Big |P_{{{\mathbb {E}}}_{m}}\bigg )\bigg \rangle \Big | \rho _{i}\in \mathbb {Z} \Bigg \}, \end{aligned}$$

where $h_{{{\mathbb {E}}}}\Big (r_{{{\mathbb {E}}}_{m}}(\rho _{i})e^{2\pi i \psi _{{{\mathbb {E}}}_{m}}(\rho _{i})}\big |P_{{{\mathbb {E}}}_{m}}\Big )$ is the collection of finite complex elements denoting the complex hesitant fuzzy information along probabilities to the set ${{\mathbb {E}}}, r_{{{\mathbb {E}}}_{m}}(\rho _{i})\in [0,1]$ and $\psi _{{{\mathbb {E}}}_{m}}(\rho _{i}) \in [0, 1], m = 1, 2,...,l$ where *l* is the number of possible elements in $h_{{{\mathbb {E}}}}\Big (r_{{{\mathbb {E}}}_{m}}(\rho _{i})e^{2\pi i \psi _{{{\mathbb {E}}}_{m}}(\rho _{i})}\big |P_{{{\mathbb {E}}}_{m}}\Big ),$ $P_{{{\mathbb {E}}}_{m}}\in [0, 1]$ is the complex hesitant probability of $r_{{{\mathbb {E}}}_{m}}(\rho _{i})e^{2\pi i \psi _{{{\mathbb {E}}}_{m}}(\rho _{i})}$ and $\sum \limits _{m} P_{{{\mathbb {E}}}_{m}}= 1.$

### Definition 2.6

Let $\mathbb {Z}$ be a universal set and *H* be the set of parameters, for any non-empty set $\mathbb {B} \subseteq H$. A pair $({{\mathbb {F}}},\mathbb {B})$ is called soft set over $\mathbb {Z}$ if there exist a mapping ${{\mathbb {F}}} : \mathbb {B} \rightarrow \mathbb {P}(\mathbb {Z})$ where $\mathbb {P}(\mathbb {Z})$ indicates the power set of $\mathbb {Z}$.

Thus, the soft set is a parametric family of the subsets of universal set. For each $b_{j}\in \mathbb {B}$, we can denote ${{\mathbb {F}}}(b_{j})$ as a subset of universal set $\mathbb {Z}$. We can also consider ${{\mathbb {F}}}(b_{j})$ as a mapping ${{\mathbb {F}}}(b_{j}): \mathbb {Z}\rightarrow \{0,1\}$ and then ${{\mathbb {F}}}(b_{j})(\rho _{i}) = 1$ equivalent to $\rho _{i}\in {{\mathbb {F}}}(b_{j})$, otherwise ${{\mathbb {F}}}(b_{j})(\rho _{i}) = 0$. Molodtsov considered many examples in Ref.^27^ to illustrate the soft set.

### Definition 2.7

Let $\mathbb {Z}$ be a universal set and *H* be the set of parameters, for any non-empty set $\mathbb {B} \subseteq H$. A pair $({{\mathbb {G}}},\mathbb {B})$ is called fuzzy soft set over $\mathbb {Z}$ if there exist a mapping ${{\mathbb {G}}}: \mathbb {B}\rightarrow \mathbb {I}(\mathbb {Z})$ where $\mathbb {I}(\mathbb {Z})$ indicates the fuzzy power set of $\mathbb {Z}$(all possible fuzzy subsets of $\mathbb {Z}$)^52^.

### Definition 2.8

Let $\mathbb {Z}$ be the universal set and $\mathbb {I}(\mathbb {Z})$ indicates the set of all fuzzy subsets of $\mathbb {Z}$ and let $R = \{0, 1, 2,..., N-1\}$ be a set of ordered grades where $N\in \{2, 3, 4,...\}$ and *H* be the set of parameters, for any non-empty set $\mathbb {B}\subseteq H$. A triple $({{\mathbb {H}}}, \mathbb {B},N)$ is called Fuzzy N-soft set over $\mathbb {Z}$ if there exist a mapping ${{\mathbb {H}}}: \mathbb {B}\rightarrow \mathbb {I}(\mathbb {Z})\times R$, with the property that for each $b_{j} \in \mathbb {B}$ there exist a unique $(\rho _{i},\mu ( \rho _{i}), s_{ij}) \in (\mathbb {I}(\mathbb {Z})\times R)$ such that $(\rho _{i},\mu ( \rho _{i}, s_{ij}) \in {{\mathbb {H}}}(b_{j}),~ b_{j}\in \mathbb {B}, ~\rho _{i} \in \mathbb {Z}~ and ~s_{ij} \in R$, where $\mathbb {I}(\mathbb {Z})\times R$ is the collection of all fuzzy soft sets over $\mathbb {Z}\times R$^39^.

### Example 2.9

Let $\mathbb {Z} = \{ \rho _{1}, \rho _{2}, \rho _{3}\}$ be the set of laptops, $H = \{b_{1}, b_{2}, b_{3},b_{4}, b_{5}\}$ be the set of parameters for the evaluations of laptop by its features, and $\mathbb {B}\subseteq H$ such that $\mathbb {B} = \{b_{1}=storage, b_{2}=graphics,b_{3}=processor\}$ and let $R = \{0,1,2,3,4\}$ be the set of grade evaluation. Then, $({{\mathbb {H}}}, \mathbb {B},5)$ is the fuzzy 5-soft set as follows:

$$\begin{aligned} {{\mathbb {H}}}(b_{1})= & {} \Big \{(\rho _{1}, 0.3, 3), (\rho _{2}, 0.5, 1), (\rho _{3}, 0.2, 2)\Big \}, \\ {{\mathbb {H}}}(b_{2})= & {} \Big \{(\rho _{1}, 0.7, 2), (\rho _{2}, 0.2, 3), (\rho _{3}, 0.4, 4)\Big \}, \\ {{\mathbb {H}}}(b_{3})= & {} \Big \{(\rho _{1}, 0.2, 0), (\rho _{2}, 0.6, 4), (\rho _{3}, 0.8, 1)\Big \}. \end{aligned}$$

It can also be represented in tabular form as follow:

**Table:** 

$({{\mathbb {H}}},\mathbb {B},5)$	$b_{1}$	$b_{3}$	$b_{3}$
$\rho _{1}$	(0.3, 3)	(0.7, 2)	(0.2, 0)
$\rho _{2}$	(0.5, 1)	(0.2, 3)	(0.6, 4)
$\rho _{3}$	(0.2, 2)	(0.4, 4)	(0.8, 1)

For interpretation, the above table is of 5-soft set $({{\mathbb {H}}}, \mathbb {B},5)$ created on the laptop’s storage, graphics, and processor. Where, in the bottom middle cell 4 is the ordered grade $(s_{32})$ of the laptop $\rho _{3}$ with respect to $b_{2}$ = graphics , Similarly, in the top-right cell 0, is the laptop’s ordered grade $(s_{13})$ of the laptop $\rho _{1}$ with respect to $b_{3}$ = processor. In this case, a grade of 0 does not indicate that the evaluation was insufficient or that the information was inadequate.

## Complex probabilistic hesitant fuzzy N-soft set

We created a model of a complex probabilistic hesitant fuzzy N-soft set (CPHFNSS) as stated in the introduction, and we used some numerical examples to show how it works. The complex probabilistic hesitant fuzzy N-soft set (CPHFNSS) framework controls uncertainty, ambiguity, and imprecision in decision-making domains, making it important. An increasingly complex and data-driven world, CPHFNSS provides a diverse and powerful tool to simulate and evaluate real-life events. Its capacity to mix probabilistic, hesitant, and fuzzy information helps decision-makers grasp diverse challenges and make more accurate and robust decisions. The aggregation operations are covered along with some fundamental operations. Also, prove their fundamental rules as well. The mathematical examples have also been resolved in order to demonstrate the validity and competency of the research work in this section.

### Definition 3.1

(^53^) Let $\mathbb {Z}$ be a universal set and *H* be the set of parameters, for any non-empty set $\mathbb {B} \subseteq H$and let $R = \{0, 1, 2, ..., N-1\}$ be a set of ordered grades where $N\in \{2, 3, 4, 5, ...\}$. A triple $({{\mathbb {I}}},\mathbb {B}, N)$ is called complex probabilistic hesitant fuzzy N-soft set over $\mathbb {Z}$ if there exist a mapping ${{\mathbb {I}}}: \mathbb {B}\rightarrow \mathbb {I}(\mathbb {Z})\times R$ with the property that for each $b_{j} \in \mathbb {B}$ there exist a unique $(\rho _{i}, s_{ij}) \in (\mathbb {Z} \times R)$ such that $((\rho _{i}, s_{ij}),h_{{{\mathbb {I}}}} )\in {{\mathbb {I}}}(b_{j}),~ b_{j}\in \mathbb {B}, ~\rho _{i} \in \mathbb {Z}~ and ~s_{ij} \in R$, where $\mathbb {I}(\mathbb {Z})\times R$ indicates the all possible complex probabilistic hesitant fuzzy subset of $\mathbb {Z}\times R$. It is expressed as:

$$\begin{aligned} {{\mathbb {I}}}(b_{j}) = \Bigg \{\bigg \langle ( \rho _{i}, s_{ij}),h_{{{\mathbb {I}}}}\bigg (r_{{{\mathbb {I}}}_{m}}( \rho _{i}, s_{ij})e^{2\pi i\psi _{{{\mathbb {I}}}_{m}}( \rho _{i}, s_{ij})}\Big |P_{{{\mathbb {I}}}_{m}}\bigg )\bigg \rangle \Big | ( \rho _{i}, s_{ij})\in \mathbb {Z}\times R \Bigg \}, ~\forall b_{j}\in \mathbb {B}\subseteq H, \end{aligned}$$

where $h_{{{\mathbb {I}}}}\Big (r_{{{\mathbb {I}}}_{m}}(\rho _{i}, s_{ij})e^{2\pi i \psi _{{{\mathbb {I}}}_{m}}(\rho _{i}, s_{ij})}\big |P_{{{\mathbb {I}}}_{m}}\Big )$ is the collection of complex elements representing the complex hesitant fuzzy information along probabilities to the set ${{\mathbb {I}}}, r_{{{\mathbb {I}}}_{m}}(\rho _{i}, s_{ij})\in [0,1]$ and $\psi _{{{\mathbb {I}}}_{m}}(\rho _{i}, s_{ij}) \in [0, 1], m = 1, 2,...,l$ where *l* is the number of possible elements in $h_{{{\mathbb {I}}}}\Big (r_{{{\mathbb {I}}}_{m}}(\rho _{i}, s_{ij}) e^{2\pi i \psi _{{{\mathbb {I}}}_{m}}(\rho _{i}, s_{ij}) }\big |P_{{{\mathbb {I}}}_{m}}\Big ),$ $P_{{{\mathbb {I}}}_{m}}\in [0, 1]$ is the complex hesitant probability of $r_{{{\mathbb {I}}}_{m}}(\rho _{i}, s_{ij}) e^{2\pi i \psi _{{{\mathbb {I}}}_{m}}(\rho _{i}, s_{ij}) }$ and $\sum \limits _{m} P_{{{\mathbb {I}}}_{m}}= 1.$

### Example 3.2

The evaluation of a lecturer at a university is based on the star ratings and grades given by a selection board, which consists of the vice chancellor, subject specialist, chairman, and psychologist. Let $\mathbb {Z} = \{ \rho _{1}, \rho _{2}, \rho _{3}\}$ be the set of candidates attending a university interview and $\mathbb {B}\subseteq H$ be the set of parameters for the evaluation, by the selection board, such that $\mathbb {B}= \big \{b_{1} = Experience, b_{2}= interpersonal~skills, b_{3}= Attiude ~of~ answering~ the ~questions\big \}$. Table 1 can be used to create a 5-soft set, where four stars indicate Excellent, three stars indicate very good, two stars indicate good, one star indicates satisfactory and hole indicates unsatisfactory.

This star-rating is easily identifiable by the numbers for example 0 represents $\circ$, 1 represents $\star$, 2 represents $\star \star$, 3 represents $\star \star \star$, 4 represents $\star \star \star \star$ and we can be used to create 5-soft set, as represented in Table 2.

It is sufficient when this information is accurately and unambiguously extracted from actual data. It has to do with the N-Soft Set. However, if the assessments are ambiguous, hesitant, and random, we might need to use CPHFNSS, which gives us more flexibility in determining how these grades are assigned to applicants. For this reason, the following CPHFNSS is defined. Then, $({{\mathbb {I}}},\mathbb {B}, 5)$ is the complex probabilistic hesitant fuzzy 5-soft set, represented in Table 3.

**Table 1:** Star evaluation by the selection board.

$\mathbb {Z} /\mathbb {B}$	$b_{1}$	$b_{2}$	$b_{3}$
$\rho _{1}$	$\star \star$	$\star \star \star$	$\star \star$
$\rho _{2}$	$\circ$	$\star \star \star \star$	$\star \star \star$
$\rho _{3}$	$\star \star \star \star \star$	$\star$	$\star \star$

**Table 2:** Five-soft set from Table 1.

$(A,\mathbb {B},6)$	$b_{1}$	$b_{2}$	$b_{3}$
$\rho _{1}$	2	3	2
$\rho _{2}$	0	4	3
$\rho _{3}$	5	1	2

**Table 3:** Tabular representation of CPHF 5-SS.

$({{\mathbb {I}}},\mathbb {B},5)$	$b_{1}$	$b_{2}$	$b_{3}$
$\rho _{1}$	$$\left( 2, \left\{ \begin{array}{l} 0.7e^{2\pi i 0.4}|0.2,\\ 0.8e^{2\pi i 0.8}|0.8 \end{array} \right\} \right)$$	$$\left( 3, \left\{ \begin{array}{l} 0.9e^{2\pi i 0.8}|0.2,\\ 0.4e^{2\pi i 0.2}|0.3,\\ 0.8e^{2\pi i 0.1}|0.5 \end{array} \right\} \right)$$	$$\left( 2, \left\{ \begin{array}{l} 0.8e^{2\pi i 0.6}|0.4,\\ 0.7e^{2\pi i 0.2}|0.6 \end{array} \right\} \right)$$
$\rho _{2}$	$$\left( 0, \left\{ \begin{array}{l} 0.3e^{2\pi i 0.7}|0.2,\\ 0.2e^{2\pi i 0.4}|0.2,\\ 0.5e^{2\pi i 0.3}|0.6 \end{array} \right\} \right)$$	$$\left( 4, \left\{ \begin{array}{l} 0.9e^{2\pi i 0.6}|1 \end{array} \right\} \right)$$	$$\left( 3, \left\{ \begin{array}{l} 0.4e^{2\pi i 0.9}|0.1,\\ 0.1e^{2\pi i 0.6}|0.9 \end{array} \right\} \right)$$
$\rho _{3}$	$$\left( 2, \left\{ \begin{array}{l} 0.1e^{2\pi i 0.7}|1 \end{array} \right\} \right)$$	$$\left( 1, \left\{ \begin{array}{l} 0.5e^{2\pi i 0.3}|1 \end{array} \right\} \right)$$	$$\left( 2, \left\{ \begin{array}{l} 0.7e^{2\pi i 0.4}|0.1,\\ 0.4e^{2\pi i 0.3}|0.2,\\ 0.9e^{2\pi i 0.8}|0.7 \end{array} \right\} \right)$$

### Definition 3.3

(^53^) Let $\mathbb {Z}$ be a universal set and *H* be the set of parameters,there exist non-empty set $\mathbb {B} \subseteq H$. A triple $({{\mathbb {J}}},\mathbb {B},N)$ is called empty CPHFNSS over $\mathbb {Z}$. If ${{\mathbb {J}}}(b_{j}) = \phi$ for all $b_{j}\in \mathbb {B}.$

### Definition 3.4

(^53^) Let $\mathbb {Z}$ be a universal set and *H* be the set of parameters, there exist non-empty set $\mathbb {B} \subseteq H$. A triple$({{\mathbb {K}}},\mathbb {B},N)$ is called full CPHFNSS over $\mathbb {Z}$. If ${{\mathbb {K}}}(b_{j}) = 1$ for all $b_{j}\in \mathbb {B}.$

### Fundamental operations of CPHFNSS

In this subsection we will develop the fundamental operations for CPHFNSS such as extended union and intersection, restricted union and intersection, weak complement, as well as top and bottom weak complement with the numerical examples. We also defined its several properties.

#### Definition 3.5

(^53^) Let $\mathbb {Z}$ be a universal set. $({{\mathbb {I}}_{1}},\mathbb {B}_{1}, N_{1})$ and $({{\mathbb {I}}_{2}},\mathbb {B}_{2}, N_{2})$ are two CPHFNSS over $\mathbb {Z}$ then the restricted union is described as:

$$\begin{aligned} ({{\mathbb {L}}},\mathbb {E}, \mathbb {K}) = ({{\mathbb {I}}_{1}},\mathbb {B}_{1}, N_{1}) \Cup ({{\mathbb {I}}_{2}},\mathbb {B}_{2}, N_{2}), \end{aligned}$$

where ${{\mathbb {L}}} = {{\mathbb {I}}_{1}} \Cup {{\mathbb {I}}_{2}}$, $\mathbb {E} = \mathbb {B}_{1} \cap \mathbb {B}_{2} \ne \phi$ and $\mathbb {K} = max(N_{1},N_{2})$; $\forall e_{j}\in \mathbb {E}$ and $\rho _{i} \in \mathbb {Z}$,

$$\bigg \langle (\rho _{i},s_{ij}),h_{{{\mathbb {L}}}}\bigg (r_{{{\mathbb {L}}}_{m}}(\rho _{i}, s_{ij})e^{2\pi i\psi _{{{\mathbb {L}}}_{m}}(\rho _{i}, s_{ij})}\Big |P_{{{\mathbb {L}}}_{m}}\bigg )\bigg \rangle \in {{\mathbb {L}}}(e_{j}) \Longleftrightarrow$$ $$s_{ij} = max(s_{ij}^{\shortmid }, s_{ij}^{\parallel })$$ and

$$\begin{aligned}{} & {} r_{{{\mathbb {L}}}_{m}}(\rho _{i}, s_{ij})e^{2\pi i\psi _{{{\mathbb {L}}}_{m}}(\rho _{i}, s_{ij})}\Big |P_{{{\mathbb {L}}}_{m}}\\{} & {} \quad = \left\{ \begin{array}{ll} \ r_{{{\mathbb {I}}_{1}}_{m}}(\rho _{i},s_{ij}^{\shortmid })e^{2\pi i\psi _{{{\mathbb {I}}_{1}}_{m}}(\rho _{i},s_{ij}^{\shortmid })}\Big |P_{{{\mathbb {I}}_{1}}_{m}} &{},~ {\text { if}} ~~m \in h_{{{\mathbb {I}}_{1}}_{m}}-h_{{{\mathbb {I}}_{2}}_{m}},\\ ~ r_{{{\mathbb {I}}_{2}}_{m}}(\rho _{i},s_{ij}^{\parallel })e^{2\pi i\psi _{{{\mathbb {I}}_{2}}_{m}}(\rho _{i},s_{ij}^{\parallel })}\Big |P_{{{\mathbb {I}}_{2}}_{m}} &{}, ~ {\text { if}} ~~m \in h_{{{\mathbb {I}}_{2}}_{m}}-h_{{{\mathbb {I}}_{1}}_{m}}, \\ ~ max\Big \{r_{{{\mathbb {I}}_{1}}_{m}}(\rho _{i},s_{ij}^{\shortmid }),r_{{{\mathbb {I}}_{2}}_{m}}(\rho _{i},s_{ij}^{\parallel })\Big \}e^{2\pi i max\Big \{\psi _{{{\mathbb {I}}_{1}}_{m}}(\rho _{i},s_{ij}^{\shortmid }),\psi _{{{\mathbb {I}}_{2}}_{m}}(\rho _{i},s_{ij}^{\parallel })\Big \}} \Big |P_{{{\mathbb {I}}_{1}}_{m}} \cdot P_{{{\mathbb {I}}_{2}}_{m}} &{}, ~{\text { if}} ~~m \in h_{{{\mathbb {I}}_{1}}_{m}}\cap h_{{{\mathbb {I}}_{2}}_{m}}, \end{array} \right. \end{aligned}$$

with $s_{ij}^{\shortmid } \in {{\mathbb {I}}_{1}}(b_{j}^{1})$ and $s_{ij}^{\parallel } \in {{\mathbb {I}}_{2}}(b_{j}^{2})$ while $b_{j}^{1} \in \mathbb {B}_{1}$ and $b_{j}^{2}\in \mathbb {B}_{2}$.

#### Definition 3.6

(^53^) Let $\mathbb {Z}$ be a universal set. $({{\mathbb {I}}_{1}},\mathbb {B}_{1}, N_{1})$ and $({{\mathbb {I}}_{2}},\mathbb {B}_{2}, N_{2})$ are two CPHFNSS over $\mathbb {Z}$ then the extended union is described as:

$$\begin{aligned} ({{\mathbb {M}}},\mathbb {F}, \mathbb {K}) = ({{\mathbb {I}}_{1}},\mathbb {B}_{1}, N_{1}) \sqcup ({{\mathbb {I}}_{2}},\mathbb {B}_{2}, N_{2}), \end{aligned}$$

where ${{\mathbb {M}}} = {{\mathbb {I}}_{1}} \sqcup {{\mathbb {I}}_{2}}$, $\mathbb {F} = \mathbb {B}_{1} \cup \mathbb {B}_{2}$ and $\mathbb {K} = max(N_{1},N_{2})$; $\forall f_{j}\in \mathbb {F}$ and $\rho _{i} \in \mathbb {Z}$, with $f_{j}^{1}\in \mathbb {B}_{1}$ and $f_{j}^{2}\in \mathbb {B}_{2}.$

$$\begin{aligned} \ {{\mathbb {M}}}(f_{j}) = \left\{ \begin{array}{ll} \ {{\mathbb {I}}_{1}}(f_{j}^{1}) &{},~ {\text { if}} ~~f_{j} \in \mathbb {B}_{1}-\mathbb {B}_{2},\\ ~{{\mathbb {I}}_{2}}(f_{j}^{2}) &{}, ~ {\text { if}} ~~f_{j} \in \mathbb {B}_{2}-\mathbb {B}_{1}, \\ ~{{\mathbb {I}}_{1}}(f_{j}^{1}) \Cup {{\mathbb {I}}_{2}}(f_{j}^{2}) &{}, ~{\text { if}} ~~f_{j} \in \mathbb {B}_{1}\cap \mathbb {B}_{2}. \end{array} \right. \end{aligned}$$

#### Definition 3.7

(^53^) Let $\mathbb {Z}$ be a universal set. $({{\mathbb {I}}_{1}},\mathbb {B}_{1}, N_{1})$ and $({{\mathbb {I}}_{2}},\mathbb {B}_{2}, N_{2})$ are two CPHFNSS over $\mathbb {Z}$ then the restricted intersection is described as:

$$\begin{aligned} ({{\mathbb {N}}},\mathbb {E}, \mathbb {J}) = ({{\mathbb {I}}_{1}},\mathbb {B}_{1}, N_{1}) \Cap ({{\mathbb {I}}_{2}},\mathbb {B}_{2}, N_{2}), \end{aligned}$$

where ${{\mathbb {N}}} = {{\mathbb {I}}_{1}} \Cap {{\mathbb {I}}_{2}}$, $\mathbb {E} = \mathbb {B}_{1} \cap \mathbb {B}_{2} \ne \phi$ and $\mathbb {J} = min(N_{1},N_{2})$; $\forall e_{j}\in \mathbb {E}$ and $\rho _{i} \in \mathbb {Z}$, $\bigg \langle (\rho _{i},s_{ij}),h_{{{\mathbb {N}}}}\bigg (r_{{{\mathbb {N}}}_{m}}(\rho _{i}, s_{ij})e^{2\pi i\psi _{{{\mathbb {N}}}_{m}}(\rho _{i}, s_{ij})}\Big |P_{{{\mathbb {N}}}_{m}}\bigg )\bigg \rangle \in {{\mathbb {N}}}(e_{j}) \Longleftrightarrow$ $s_{ij} = max(s_{ij}^{\shortmid }, s_{ij}^{\parallel })$ and

$$\begin{aligned}{} & {} r_{{{\mathbb {N}}}_{m}}(\rho _{i}, s_{ij})e^{2\pi i\psi _{{{\mathbb {N}}}_{m}}(\rho _{i}, s_{ij})}\Big |P_{{{\mathbb {N}}}_{m}} \\{} & {} \quad = \left\{ \begin{array}{ll} \ r_{{{\mathbb {I}}_{1}}_{m}}(\rho _{i},s_{ij}^{\shortmid })e^{2\pi i\psi _{{{\mathbb {I}}_{1}}_{m}}(\rho _{i},s_{ij}^{\shortmid })}\Big |P_{{{\mathbb {I}}_{1}}_{m}} &{},~ {\text { if}} ~~m \in h_{{{\mathbb {I}}_{1}}_{m}}-h_{{{\mathbb {I}}_{2}}_{m}},\\ ~ r_{{{\mathbb {I}}_{2}}_{m}}(\rho _{i},s_{ij}^{\parallel })e^{2\pi i\psi _{{{\mathbb {I}}_{2}}_{m}}(\rho _{i},s_{ij}^{\parallel })}\Big |P_{{{\mathbb {I}}_{2}}_{m}} &{}, ~ {\text { if}} ~~m \in h_{{{\mathbb {I}}_{2}}_{m}}-h_{{{\mathbb {I}}_{1}}_{m}}, \\ ~ min\Big \{r_{{{\mathbb {I}}_{1}}_{m}}(\rho _{i},s_{ij}^{\shortmid }),r_{{{\mathbb {I}}_{2}}_{m}}(\rho _{i},s_{ij}^{\parallel })\Big \}e^{2\pi i min\Big \{\psi _{{{\mathbb {I}}_{1}}_{m}}(\rho _{i},s_{ij}^{\shortmid }),\psi _{{{\mathbb {I}}_{2}}_{m}}(\rho _{i},s_{ij}^{\parallel })\Big \}} \Big |P_{{{\mathbb {I}}_{1}}_{m}} \cdot P_{{{\mathbb {I}}_{2}}_{m}} &{}, ~{\text { if}} ~~m \in h_{{{\mathbb {I}}_{1}}_{m}}\cap h_{{{\mathbb {I}}_{2}}_{m}}, \end{array} \right. \end{aligned}$$

with $s_{ij}^{\shortmid } \in {{\mathbb {I}}_{1}}(b_{j}^{1})$ and $s_{ij}^{\parallel } \in {{\mathbb {I}}_{2}}(b_{j}^{2})$ while $b_{j}^{1} \in \mathbb {B}_{1}$ and $b_{j}^{2}\in \mathbb {B}_{2}$.

#### Definition 3.8

(^53^) Let $\mathbb {Z}$ be a universal set. $({{\mathbb {I}}_{1}},\mathbb {B}_{1}, N_{1})$ and $({{\mathbb {I}}_{2}},\mathbb {B}_{2}, N_{2})$ are two CPHFNSS over $\mathbb {Z}$ then the extended intersection is described as:

$$\begin{aligned} ({{\mathbb {O}}},\mathbb {F}, \mathbb {K}) = ({{\mathbb {I}}_{1}},\mathbb {B}_{1}, N_{1}) \sqcap ({{\mathbb {I}}_{2}},\mathbb {B}_{2}, N_{2}), \end{aligned}$$

where ${{\mathbb {O}}} = {{\mathbb {I}}_{1}} \sqcap {{\mathbb {I}}_{2}}$, $\mathbb {F} = \mathbb {B}_{1} \cup \mathbb {B}_{2}$ and $\mathbb {K} = max(N_{1},N_{2})$; $\forall f_{j}\in \mathbb {F}$ and $\rho _{i} \in \mathbb {Z}$, with $f_{j}^{1}\in \mathbb {B}_{1}$ and $f_{j}^{2}\in \mathbb {B}_{2}.$

$$\begin{aligned} \ {{\mathbb {O}}}(f_{j}) = \left\{ \begin{array}{ll} \ {{\mathbb {I}}_{1}}(f_{j}^{1}) &{},~ {\text { if}} ~~f_{j} \in \mathbb {B}_{1}-\mathbb {B}_{2},\\ ~{{\mathbb {I}}_{2}}(f_{j}^{2}) &{}, ~ {\text { if}} ~~f_{j} \in \mathbb {B}_{2}-\mathbb {B}_{1}, \\ ~{{\mathbb {I}}_{1}}(f_{j}^{1}) \Cap {{\mathbb {I}}_{2}}(f_{j}^{2}) &{}, ~{\text { if}} ~~f_{j} \in \mathbb {B}_{1}\cap \mathbb {B}_{2}. \end{array} \right. \end{aligned}$$

#### Definition 3.9

(^53^) Let $\mathbb {Z}$ be a universal set. $({{\mathbb {I}}},\mathbb {B}, N)$ is the CPHFNSS over $\mathbb {Z}$ then weak CPHFNSS complement is represented by $({{\mathbb {I}}}^{c},\mathbb {B}, N)$ where ${{\mathbb {I}}}^{c}(b_{j}) \cap {{\mathbb {I}}}(b_{j}) = \Phi ;~\forall b_{j}\in \mathbb {B}$ and it is defined as:

$$\begin{aligned} {{\mathbb {I}}}^{c}(b_{j}) = \Bigg \{\bigg \langle (\rho _{i}, s_{ij}), h_{{{{\mathbb {I}}}}^{c}}\bigg (\big (1 - r_{{{\mathbb {I}}}_{m}}(\rho _{i}, s_{ij})\big )e^{2\pi i\big (1 - \psi _{{{\mathbb {I}}}_{m}}(\rho _{i}, s_{ij})\big )}\Big |1 - P_{{{\mathbb {I}}}_{m}}\bigg )\bigg \rangle \Big | (\rho _{i}, s_{ij})\in \mathbb {Z} \times R \Bigg \}, ~\forall b_{j}\in \mathbb {B}\subseteq H. \end{aligned}$$

#### Definition 3.10

(^53^) Let $\mathbb {Z}$ be a universal set. For any CHFNSS $({{\mathbb {I}}},\mathbb {B}, N)$ over $\mathbb {Z}$ then bottom weak CPHFNSS complement is represented by $({{\mathbb {I}}}^{\preccurlyeq },\mathbb {B}, N)$; $\forall b_{j}\in \mathbb {B}$ and it is defined as:

$$\begin{aligned} {{\mathbb {I}}}^{\preccurlyeq }(b_{j})= \left\{ \begin{array}{ll} (\rho _{i}, 0), h_{{{{\mathbb {I}}}}^{\preccurlyeq }}\bigg (\big (1 - r_{{{\mathbb {I}}}_{m}}(\rho _{i}, s_{ij})\big )e^{2\pi i\big (1 - \psi _{{{\mathbb {I}}}_{m}}(\rho _{i}, s_{ij})\big )}\Big |1 - P_{{{\mathbb {I}}}_{m}}\bigg )~~~~~~~,{\text {if}} ~~s_{ij}~>~0,\\ (\rho _{i}, N - 1), h_{{{{\mathbb {I}}}}^{\preccurlyeq }}\bigg (\big (1 - r_{{{\mathbb {I}}}_{m}}(\rho _{i}, s_{ij})\big )e^{2\pi i\big (1 - \psi _{{{\mathbb {I}}}_{m}}(\rho _{i}, s_{ij})\big )}\Big |1 - P_{{{\mathbb {I}}}_{m}}\bigg )~,{\text {if}} ~~ s_{ij}~=~0. \end{array} \right. \end{aligned}$$

#### Definition 3.11

(^53^) Let $\mathbb {Z}$ be a universal set. For any CHFNSS $({{\mathbb {I}}},\mathbb {B}, N)$ over $\mathbb {Z}$ then top weak CPHFNSS complement is represented by $({{\mathbb {I}}}^{\curlyeqprec },\mathbb {B}, N)$; $\forall b_{j}\in \mathbb {B}$ and it is defined as:

$$\begin{aligned} {{\mathbb {I}}}^{\curlyeqprec }(b_{j})= \left\{ \begin{array}{ll} (\rho _{i}, N - 1), h_{{{{\mathbb {I}}}}^{\curlyeqprec }}\bigg (\big (1 - r_{{{\mathbb {I}}}_{m}}(\rho _{i}, s_{ij})\big )e^{2\pi i\big (1 - \psi _{{{\mathbb {I}}}_{m}}(\rho _{i}, s_{ij})\big )}\Big |1 - P_{{{\mathbb {I}}}_{m}}\bigg )~,{\text {if}} ~~s_{ij}<~N - 1,\\ (\rho _{i}, 0), h_{{{{\mathbb {I}}}}^{\curlyeqprec }}\bigg (\big (1 - r_{{{\mathbb {I}}}_{m}}(\rho _{i}, s_{ij})\big )e^{2\pi i\big (1 - \psi _{{{\mathbb {I}}}_{m}}(\rho _{i}, s_{ij})\big )}\Big |1 - P_{{{\mathbb {I}}}_{m}}\bigg )~~~~~~~,{\text {if}} ~~ s_{ij}=~N - 1. \end{array}\right. \end{aligned}$$

#### Proposition 3.12

Given that $({{\mathbb {I}}_{1}},\mathbb {B}_{1}, N_{1})$, $({{\mathbb {I}}_{2}},\mathbb {B}_{2}, N_{2})$ and $({{\mathbb {I}}_{3}},\mathbb {B}_{3}, N_{3})$ are any three CPHFNSS on $\mathbb {Z}$, then the following laws hold.

Idempotent Laws:

i. $$({{\mathbb {I}}_{1}},\mathbb {B}_{1}, N_{1})\Cup ({{\mathbb {I}}_{1}},\mathbb {B}_{1}, N_{1}) = ({{\mathbb {I}}_{1}},\mathbb {B}_{1}, N_{1}).$$
ii. $$({{\mathbb {I}}_{1}},\mathbb {B}_{1}, N_{1})\sqcup ({{\mathbb {I}}_{1}},\mathbb {B}_{1}, N_{1}) = ({{\mathbb {I}}_{1}},\mathbb {B}_{1}, N_{1}).$$
iii. $$({{\mathbb {I}}_{1}},\mathbb {B}_{1}, N_{1})\Cap ({{\mathbb {I}}_{1}},\mathbb {B}_{1}, N_{1}) = ({{\mathbb {I}}_{1}},\mathbb {B}_{1}, N_{1}).$$
iv. $$({{\mathbb {I}}_{1}},\mathbb {B}_{1}, N_{1})\sqcap ({{\mathbb {I}}_{1}},\mathbb {B}_{1}, N_{1}) = ({{\mathbb {I}}_{1}},\mathbb {B}_{1}, N_{1}).$$

Commutative Laws:

xxii. $$({{\mathbb {I}}_{1}},\mathbb {B}_{1}, N_{1})\Cup ({{\mathbb {I}}_{2}},\mathbb {B}_{2}, N_{2}) = ({{\mathbb {I}}_{2}},\mathbb {B}_{2}, N_{2})\Cup ({{\mathbb {I}}_{1}},\mathbb {B}_{1}, N_{1})$$.
xxiii. $$({{\mathbb {I}}_{1}},\mathbb {B}_{1}, N_{1})\sqcup ({{\mathbb {I}}_{2}},\mathbb {B}_{2}, N_{2}) = ({{\mathbb {I}}_{2}},\mathbb {B}_{2}, N_{2})\sqcup ({{\mathbb {I}}_{1}},\mathbb {B}_{1}, N_{1})$$.
xxiv. $$({{\mathbb {I}}_{1}},\mathbb {B}_{1}, N_{1})\Cap ({{\mathbb {I}}_{2}},\mathbb {B}_{2}, N_{2}) = ({{\mathbb {I}}_{2}},\mathbb {B}_{2}, N_{2})\Cap ({{\mathbb {I}}_{1}},\mathbb {B}_{1}, N_{1})$$.
xxv. $$({{\mathbb {I}}_{1}},\mathbb {B}_{1}, N_{1})\sqcap ({{\mathbb {I}}_{2}},\mathbb {B}_{2}, N_{2}) = ({{\mathbb {I}}_{2}},\mathbb {B}_{2}, N_{2})\sqcap ({{\mathbb {I}}_{1}},\mathbb {B}_{1}, N_{1})$$.

Associative Laws:

ix. $$({{\mathbb {I}}_{1}},\mathbb {B}_{1}, N_{1})\Cup \Big (({{\mathbb {I}}_{2}},\mathbb {B}_{2}, N_{2})\Cup ({{\mathbb {I}}_{3}},\mathbb {B}_{3}, N_{3})\Big )= \Big (({{\mathbb {I}}_{1}},\mathbb {B}_{1}, N_{1})\Cup ({{\mathbb {I}}_{2}},\mathbb {B}_{2}, N_{2})\Big )\Cup ({{\mathbb {I}}_{3}},\mathbb {B}_{3}, N_{3})$$.
x. $$({{\mathbb {I}}_{1}},\mathbb {B}_{1}, N_{1})\sqcup \Big (({{\mathbb {I}}_{2}},\mathbb {B}_{2}, N_{2})\sqcup ({{\mathbb {I}}_{3}},\mathbb {B}_{3}, N_{3})\Big )= \Big (({{\mathbb {I}}_{1}},\mathbb {B}_{1}, N_{1})\sqcup ({{\mathbb {I}}_{2}},\mathbb {B}_{2}, N_{2})\Big )\sqcup ({{\mathbb {I}}_{3}},\mathbb {B}_{3}, N_{3})$$.
xi. $$({{\mathbb {I}}_{1}},\mathbb {B}_{1}, N_{1})\Cap \Big (({{\mathbb {I}}_{2}},\mathbb {B}_{2}, N_{2})\Cap ({{\mathbb {I}}_{3}},\mathbb {B}_{3}, N_{3})\Big )= \Big (({{\mathbb {I}}_{1}},\mathbb {B}_{1}, N_{1})\Cap ({{\mathbb {I}}_{2}},\mathbb {B}_{2}, N_{2})\Big )\Cap ({{\mathbb {I}}_{3}},\mathbb {B}_{3}, N_{3})$$.
xii. $$({{\mathbb {I}}_{1}},\mathbb {B}_{1}, N_{1})\sqcap \Big (({{\mathbb {I}}_{2}},\mathbb {B}_{2}, N_{2})\sqcap ({{\mathbb {I}}_{3}},\mathbb {B}_{3}, N_{3})\Big )= \Big (({{\mathbb {I}}_{1}},\mathbb {B}_{1}, N_{1})\sqcap ({{\mathbb {I}}_{2}},\mathbb {B}_{2}, N_{2})\Big )\sqcap ({{\mathbb {I}}_{3}},\mathbb {B}_{3}, N_{3})$$.

#### Proposition 3.13

Given that $({{\mathbb {I}}_{1}},\mathbb {B}_{1}, N_{1})$ and $({{\mathbb {I}}_{2}},\mathbb {B}_{2}, N_{2})$ are any two CPHFSS on $\mathbb {Z}$, then the following laws hold.

Involution Law:

i. $$\bigg (\Big ({{\mathbb {I}}_{1}}^{c}\Big )^{c},\mathbb {B}_{1}, N_{1}\bigg )= ({{\mathbb {I}}_{1}},\mathbb {B}_{1}, N_{1})$$.

De-Morgan’s Laws:

ii. $$\Big ({{\mathbb {I}}_{1}}^{c},\mathbb {B}_{1}, N_{1}\Big )\Cap \Big ({{\mathbb {I}}_{2}}^{c},\mathbb {B}_{2}, N_{2}\Big ) = \Big (\big ({{\mathbb {I}}_{1}}\Cup {{\mathbb {I}}_{2}}\big )^{c}, (\mathbb {B}_{1}\cap \mathbb {B}_{2}), min(N_{1},N_{2}) \Big )$$.
iii. $$\Big ({{\mathbb {I}}_{1}}^{c},\mathbb {B}_{1}, N_{1}\Big )\sqcap \Big ({{\mathbb {I}}_{2}}^{c},\mathbb {B}_{2}, N_{2}\Big ) = \Big (\big ({{\mathbb {I}}_{1}}\sqcup {{\mathbb {I}}_{2}}\big )^{c}, (\mathbb {B}_{1}\cup \mathbb {B}_{2}), max(N_{1},N_{2}) \Big )$$.
iv. $$\Big ({{\mathbb {I}}_{1}}^{c},\mathbb {B}_{1}, N_{1}\Big )\Cup \Big ({{\mathbb {I}}_{2}}^{c},\mathbb {B}_{2}, N_{2}\Big ) = \Big (\big ({{\mathbb {I}}_{1}}\Cap {{\mathbb {I}}_{2}}\big )^{c}, (\mathbb {B}_{1}\cap \mathbb {B}_{2}), max(N_{1},N_{2}) \Big )$$.
v. $$\Big ({{\mathbb {I}}_{1}}^{c},\mathbb {B}_{1}, N_{1}\Big )\sqcup \Big ({{\mathbb {I}}_{2}}^{c},\mathbb {B}_{2}, N_{2}\Big ) = \Big (\big ({{\mathbb {I}}_{1}}\sqcap {{\mathbb {I}}_{2}}\big )^{c}, (\mathbb {B}_{1}\cup \mathbb {B}_{2}), max(N_{1},N_{2}) \Big )$$.

The score function in the complex probabilistic hesitant fuzzy N-soft set framework enhances its relevance and usefulness. A scoring mechanism is established with the aim of resolving various comparability concerns. This function quantifies the appropriateness or preference of choice alternatives, making it easier to rank and acquire solutions in complicated decision situations.

#### Definition 3.14

The score function $\eth$ for a CPHFNS element ${\mathbb {I}}$ is defined as:

$$\begin{aligned} \eth ({\mathbb {I}}) = \left(\frac{max_{j=1}^{s}s_{ij}}{N-1}\right)\times \frac{1}{s}\sum \limits _{j=1}^{s}\bigg [\frac{1}{l}\sum \limits _{m=1}^{l} \Big (\frac{r_{{\mathbb {I}}(b_{j})_{m}}+ \omega _{{\mathbb {I}}(b_{j})_{m}}}{2} \times P_{{\mathbb {I}}(b_{j})_{m}}\Big )\bigg ], \end{aligned}$$

where *l* is the number of feasible hesitant values and *s* is the number of parameters.

#### Remark 3.15

If we want to find the score w.r.t single parameter $b_{j}$ we will put s=j in the above Definition 3.14, such as

$$\begin{aligned} \eth ({\mathbb {I}}) = \left(\frac{s_{ij}}{N-1}\right)\times \frac{1}{l}\sum \limits _{m=1}^{l} \Big (\frac{r_{{\mathbb {I}}(b_{j})_{m}}+ \omega _{{\mathbb {I}}(b_{j})_{m}}}{2} \times P_{{\mathbb {I}}(b_{j})_{m}}\Big ). \end{aligned}$$

#### Definition 3.16

The ordered relation between CPHFNS elements ${\mathbb {I}}_{1}$ and ${\mathbb {I}}_{2}$ is defined as:

- If $$\eth ({\mathbb {I}}_{1}) > \eth ({\mathbb {I}}_{2})$$ then $${\mathbb {I}}_{1} > {\mathbb {I}}_{2}$$,
- If $$\eth ({\mathbb {I}}_{1}) < \eth ({\mathbb {I}}_{2})$$ then $${\mathbb {I}}_{1} < {\mathbb {I}}_{2}$$,
- If $\eth ({\mathbb {I}}_{1}) = \eth ({\mathbb {I}}_{2})$ then we calculate the accuracy function.

#### Definition 3.17

The accuracy function is defined as:

$$\begin{aligned} \mho ({\mathbb {I}}_{1}) = \left(\frac{s_{ij}}{N-1}\right)\times \sqrt{\frac{1}{s}\sum \limits _{j=1}^{s}\bigg [\frac{1}{l}\sum \limits _{m=1}^{l} \big (\hbar _{m} -\eth ({\mathbb {I}}_{1}) \big )^{2}}\bigg ], \end{aligned}$$

- If $$\mho ({\mathbb {I}}_{1}) > \mho ({\mathbb {I}}_{2})$$ then $${\mathbb {I}}_{1} > {\mathbb {I}}_{2}$$,
- If $$\mho ({\mathbb {I}}_{1}) < \mho ({\mathbb {I}}_{2})$$ then $${\mathbb {I}}_{1} < {\mathbb {I}}_{2}$$,
- If $$\mho ({\mathbb {I}}_{1}) = \mho ({\mathbb {I}}_{2})$$ then $${\mathbb {I}}_{1} \sim {\mathbb {I}}_{2}$$.

### Aggregation operations for CPHFNSS

In this subsection we will develop the aggregation operations for CPHFNSSs and their some verified properties. It is impossible to emphasize the significance of aggregate procedures in data analysis and decision making. These operations serve as a foundation that transforms unstructured data into actionable insights. Aggregation operations simplify complex information by condensing large volumes of data into meaningful summaries, thereby disclosing trends, patterns, and key metrics that would otherwise remain obscure. In recent decades, it have garnered a great deal of attention^25,26^. Except as otherwise specified, suppose. ${\mathbb {I}}(b_{j})=\bigg \langle (\rho _{i},s_{ij}),h_{{\mathbb {I}}}\bigg (r_{{\mathbb {I}}(b_{j})_{m}}(\rho _{i},s_{ij})e^{2\pi i\psi _{{\mathbb {I}}(b_{j})_{m}}(\rho _{i},s_{ij})}\Big |P_{{\mathbb {I}}(b_{j})_{m}}\bigg )\bigg \rangle,$ ${\mathbb {I}}(b_{j'})=\bigg \langle (\rho _{i},s_{ij'}),h_{{\mathbb {I}}}\bigg (r_{{\mathbb {I}}(b_{j'})_{m}}(\rho _{i},s_{ij'})e^{2\pi i\psi _{{\mathbb {I}}(b_{j'})_{m}}(\rho _{i},s_{ij'})}\Big |P_{{\mathbb {I}}(b_{j'})_{m}} \bigg )\bigg \rangle,$ and ${\mathbb {I}}(b_{j''})=\bigg \langle (\rho _{i},s_{ij''}),h_{{\mathbb {I}}}\bigg (r_{{\mathbb {I}}(b_{j''})_{m}}(\rho _{i},s_{ij''})e^{2\pi i\psi _{{\mathbb {I}}(b_{j''})_{m}}(\rho _{i},s_{ij''})}\Big |P_{{\mathbb {I}}(b_{j''})_{m}} \bigg )\bigg \rangle,$ are three CPHFSS and assume that

$$\begin{aligned} r_{{\mathbb {I}}(b_{j})_{m}}(\rho _{i},s_{ij})&= \phi _{j},&\psi _{{\mathbb {I}}(b_{j})_{m}}(\rho _{i},s_{ij})&= \varepsilon _{j},&P_{{\mathbb {I}}(b_{j})_{m}}&= \mathrm {P_{j}}\\ r_{{\mathbb {I}}(b_{j'})_{m}}(\rho _{i},s_{ij'})&= \phi _{j'},&\psi _{{\mathbb {I}}(b_{j'})_{m}}(\rho _{i},s_{ij'})&= \varepsilon _{j'},&P_{{\mathbb {I}}(b_{j'})_{m}}&= \mathrm {P_{j'}}\\ r_{{\mathbb {I}}(b_{j''})_{m}}(\rho _{i},s_{ij''})&= \phi _{j''},&\psi _{{\mathbb {I}}(b_{j''})_{m}}(\rho _{i},s_{ij''})&= \varepsilon _{j''},&P_{{\mathbb {I}}(b_{j''})_{m}}&= \mathrm {P_{j''}}. \end{aligned}$$

#### Definition 3.18

Let ${\mathbb {I}}(b_{j}) = \left\langle (\rho _{i},s_{ij}),h_{{\mathbb {I}}}\Big (\phi _{j}e^{2\pi i\varepsilon _{j}}\big | \mathrm {P_{j}}\Big )\right\rangle$ and ${\mathbb {I}}(b_{j'})$ = ${\langle }(\rho _{i},s_{ij'}), h_{{\mathbb {I}}}\Big (\phi _{j'}e^{2\pi i\varepsilon _{j'}}\big | \mathrm {P_{j'}}\Big ){\rangle }$ be the two CPHFNS elements and $\kappa \in \mathbb {R} > 0$ is scalar. Then the aggregation operations for CPHFNSS is defined as:

1. $${\mathbb {I}}(b_{j})^{\kappa } = \left\langle (\rho _{i},s_{ij}),h_{{\mathbb {I}}}\Big (\big (\phi _{j}\big )^{\kappa }e^{2\pi i(\varepsilon _{j})^{\kappa }}\big |\mathrm {P_{j}}\Big )\right\rangle .$$
2. $$\kappa {\mathbb {I}}(b_{j}) = \left\langle (\rho _{i},s_{ij}),h_{{\mathbb {I}}}\left(\left(1-(1 - \phi _{j})^{\kappa } \right) e^{2\pi i\left( 1 - (1 - \varepsilon _{j})^{\kappa }\right)}\big | \mathrm {P_{j}}\right) \right\rangle$$.
3. $${\mathbb {I}}(b_{j}) \otimes {\mathbb {I}}(b_{j'}) = \bigg \langle (\rho _{i},min (s_{ij},s_{ij'})), h_{{\mathbb {I}}} \Big (\big ( \phi _{j}\phi _{j'}\big )e^{2\pi i (\varepsilon _{j}\varepsilon _{j'})}\big | \mathrm {P_{j}}\mathrm {P_{j'}}\Big ){\rangle }.$$
4. $${\mathbb {I}}(b_{j}) \oplus {\mathbb {I}}(b_{j'})= {\langle } (\rho _{i}, max (s_{ij},s_{ij'})), h_{{\mathbb {I}}}\Big (\big (\phi _{j}+ \phi _{j'}- \phi _{j}\phi _{j'}\big )e^{2\pi i (\varepsilon _{j}+ \varepsilon _{j'}- \varepsilon _{j}\varepsilon _{j'})}\big |\mathrm {P_{j}}\mathrm {P_{j'}}\Big ){\rangle }$$.

#### Theorem 3.19

Let ${\mathbb {I}}(b_{j})$, ${\mathbb {I}}(b_{j'})$, and ${\mathbb {I}}(b_{j''})$ be the three complex probabilistic hesitant fuzzy soft numbers and $\kappa _{1}, \kappa _{2}, \kappa > 0$ are scalars. Then following theorems are hold:

1. $${\mathbb {I}}(b_{j}) \oplus {\mathbb {I}}(b_{j'}) = {\mathbb {I}}(b_{j'}) \oplus {\mathbb {I}}(b_{j})$$.
2. $${\mathbb {I}}(b_{j}) \otimes {\mathbb {I}}(b_{j'}) = {\mathbb {I}}(b_{j'}) \otimes {\mathbb {I}}(b_{j})$$.
3. $$\big ({\mathbb {I}}(b_{j}) \oplus {\mathbb {I}}(b_{j'})\big ) \oplus {\mathbb {I}}(b_{j''}) = {\mathbb {I}}(b_{j}) \oplus \big ({\mathbb {I}}(b_{j'}) \oplus {\mathbb {I}}(b_{j''})\big )$$.
4. $$\big ({\mathbb {I}}(b_{j}) \otimes {\mathbb {I}}(b_{j'})\big ) \otimes {\mathbb {I}}(b_{j''}) = {\mathbb {I}}(b_{j}) \otimes \big ({\mathbb {I}}(b_{j'}) \otimes {\mathbb {I}}(b_{j''})\big )$$.
5. $$\kappa \big ({\mathbb {I}}(b_{j}) \oplus {\mathbb {I}}(b_{j'})\big ) = \kappa {\mathbb {I}}(b_{j}) \oplus \kappa {\mathbb {I}}(b_{j'})$$.
6. $$\big ({\mathbb {I}}(b_{j}) \otimes {\mathbb {I}}(b_{j'})\big )^{\kappa } = {\mathbb {I}}(b_{j})^{\kappa } \otimes {\mathbb {I}}(b_{j'})^{\kappa }$$.
7. $$\kappa _{1}{\mathbb {I}}(b_{j}) \oplus \kappa _{2}{\mathbb {I}}(b_{j}) = ( \kappa _{1} + \kappa _{2}){\mathbb {I}}(b_{j})$$.
8. $${\mathbb {I}}(b_{j})^{\kappa _{1}} \otimes {\mathbb {I}}(b_{j})^{\kappa _{2}} = {\mathbb {I}}(b_{j})^{ \kappa _{1} + \kappa _{2}}$$.

The proof of (1) to (4) can easily be derived from the Definition 3.18.

(5) $$\kappa \left({\mathbb {I}}(b_{j}) \oplus {\mathbb {I}}(b_{j'})\right) = \kappa {\mathbb {I}}(b_{j}) \oplus \kappa {\mathbb {I}}(b_{j'})$$

#### Proof

  L.H.S:

$$\begin{aligned} \kappa \left({\mathbb {I}}(b_{j}) \oplus {\mathbb {I}}(b_{j'})\right) = \kappa \left\lbrace \left\langle (\rho _{i}, max (s_{ij},s_{ij'})), h_{{\mathbb {I}}}\left(\left(\phi _{j}+ \phi _{j'}- \phi _{j}\phi _{j'}\right)e^{2\pi i \left(\varepsilon _{j}+ \varepsilon _{j'}- \varepsilon _{j}\varepsilon _{j'}\right)}\big |\mathrm {P_{j}}\mathrm {P_{j'}}\right)\right\rangle \right\rbrace . \end{aligned}$$

Firstly we will multiply $\kappa$ with amplitude term.

$$\begin{aligned} \kappa \big (\phi _{j}+ \phi _{j'}- \phi _{j}\phi _{j'}\big )&= 1-\Big (1-\big (\phi _{j}+ \phi _{j'}- \phi _{j}\phi _{j'}\big )\Big )^{\kappa }\\ {}&= 1-\big (1- \phi _{j}- \phi _{j'}+ \phi _{j}\phi _{j'}\big )^{\kappa }\\ {}&= 1-\Big (\big (1- \phi _{j}\big ) - \phi _{j'}\big ( 1 - \phi _{j}\big )\Big )^{\kappa }\\ {}&= 1 - \big (\big (1- \phi _{j}\big )\big (1- \phi _{j'}\big ) \big )^{\kappa }\\ {}&= 1 - \big (1- \phi _{j}\big )^{\kappa }\big (1- \phi _{j'}\big )^{\kappa }. \end{aligned}$$

Similarly for phase term;

$$\begin{aligned} \kappa \big (\varepsilon _{j}+ \varepsilon _{j'}- \varepsilon _{j}\varepsilon _{j'}\big ) = 1 - \big (1- \varepsilon _{j}\big )^{\kappa }\big (1- \varepsilon _{j'}\big )^{\kappa }. \end{aligned}$$

This implies that

1 $$\begin{aligned} \kappa \left({\mathbb {I}}(b_{j}) \oplus {\mathbb {I}}(b_{j'})\right) = \bigg \{\bigg \langle (\rho _{i}, max (s_{ij},s_{ij'})), h_{{\mathbb {I}}}\Big (\Big ( 1 - \big (1- \phi _{j}\big )^{\kappa }\big (1- \phi _{j'}\big )^{\kappa }\Big )e^{2\pi i\left(1 - (1- \varepsilon _{j})^{\kappa }(1- \varepsilon _{j'})^{\kappa }\right)} \big | \mathrm {P_{j}}\mathrm {P_{j'}}\Big )\bigg \rangle \bigg \}. \end{aligned}$$

R.H.S:

$$\begin{aligned} \kappa {\mathbb {I}}(b_{j}) \oplus \kappa {\mathbb {I}}(b_{j'}) =&\bigg \langle (\rho _{i}, s_{ij}),h_{{\mathbb {I}}}\bigg (\Big (1-\big (1 - \phi _{j}\big )^{\kappa } \Big ) e^{2\pi i\big ( 1 - \left(1 - \varepsilon _{j}\right)^{\kappa }\big )}\big | \mathrm {P_{j}}\bigg )\bigg \rangle \oplus \\ {}&\bigg \langle (\rho _{i}, s_{ij'}),h_{{\mathbb {I}}}\bigg (\Big (1-\big (1 - \phi _{j'}\big )^{\kappa } \Big ) e^{2\pi i\big ( 1 - \left(1 - \varepsilon _{j'}\right)^{\kappa }\big )}\big |\mathrm {P_{j'}}\bigg )\bigg \rangle . \end{aligned}$$

First of all we will solve for amplitude term:

$$\begin{aligned} \Big (1-\big (1 - \phi _{j}\big )^{\kappa }\Big ) \oplus \Big (1-\big (1 - \phi _{j'}\big )^{\kappa }\Big )&= \Big (1-\big (1 - \phi _{j}\big )^{\kappa }\Big ) + \Big (1-\big (1 - \phi _{j'}\big )^{\kappa }\Big ) - \Big (1-\big (1 - \phi _{j}\big )^{\kappa }\Big )\\ {}&~~~~ \Big (1-\big (1 - \phi _{j'}\big )^{\kappa }\Big )\\ {}&= 2 - \big (1 - \phi _{j}\big )^{\kappa } -\big (1 - \phi _{j'}\big )^{\kappa } - \Big [1 - \big (1 - \phi _{j'}\big )^{\kappa } -\big (1 - \phi _{j}\big )^{\kappa } +\\ {}&~~~~ \big (1 - \phi _{j}\big )^{\kappa } \big (1 - \phi _{j'}\big )^{\kappa } \Big ]\\ {}&= 2 - \big (1 - \phi _{j}\big )^{\kappa } -\big (1 - \phi _{j'}\big )^{\kappa } - 1 + \big (1 - \phi _{j'}\big )^{\kappa } + \big (1 - \phi _{j}\big )^{\kappa } -\\ {}&~~~~ \big (1 - \phi _{j}\big )^{\kappa } \big (1 - \phi _{j'}\big )^{\kappa }\\ {}&= 1 - \big (1 - \phi _{j}\big )^{\kappa }\big (1 - \phi _{j'}\big )^{\kappa }. \end{aligned}$$

Similarly for phase term;

$$\begin{aligned} \Big (1-\big (1 - \varepsilon _{j}\big )^{\kappa }\Big ) \oplus \Big (1-\big (1 - \varepsilon _{j'}\big )^{\kappa }\Big ) = 1 - \big (1 - \varepsilon _{j}\big )^{\kappa }\big (1 - \varepsilon _{j'}\big )^{\kappa }. \end{aligned}$$

This implies that

2 $$\begin{aligned} \kappa {\mathbb {I}}(b_{j}) \oplus \kappa {\mathbb {I}}(b_{j'}) = \bigg \{\bigg \langle (\rho _{i}, max (s_{ij},s_{ij'})), h_{{\mathbb {I}}}\Big (\Big ( 1 - \big (1- \phi _{j}\big )^{\kappa }\big (1- \phi _{j'}\big )^{\kappa }\Big )e^{2\pi i \left(1 - (1- \varepsilon _{j})^{\kappa }(1- \varepsilon _{j'})^{\kappa }\right)}\big |\mathrm {P_{j}}\mathrm {P_{j'}}\Big )\bigg \rangle \bigg \}. \end{aligned}$$

From Eqs. (1) and (2);

L.H.S = R.H.S

Hence $$\kappa \Big ({\mathbb {I}}(b_{j}) \oplus {\mathbb {I}}(b_{j'})\Big ) = \kappa {\mathbb {I}}(b_{j}) \oplus \kappa {\mathbb {I}}(b_{j'})$$. $$\square$$

(6) $$\Big ({\mathbb {I}}(b_{j}) \otimes {\mathbb {I}}(b_{j'})\Big )^{\kappa } = {\mathbb {I}}(b_{j})^{\kappa } \otimes {\mathbb {I}}(b_{j'})^{\kappa }$$.

#### Proof

  L.H.S:

$$\begin{aligned} \big ({\mathbb {I}}(b_{j}) \otimes {\mathbb {I}}(b_{j'})\big )^{\kappa }&= \bigg \langle (\rho _{i},s_{ij}), h_{{\mathbb {I}}}\bigg (\Big (\phi _{j}\phi _{j'}\Big )^{\kappa }e^{2\pi i\Big (\varepsilon _{j}\varepsilon _{j'}\Big )^{\kappa }}\Big | \mathrm {P_{j}}\mathrm {P_{j'}}\bigg )\bigg \rangle \\ {}&=\bigg \langle (\rho _{i},s_{ij}), h_{{\mathbb {I}}}\bigg (\Big (\big (\phi _{j}\big )^{\kappa }\big (\phi _{j'}\big )^{\kappa }\Big )e^{2\pi i\Big (\big (\varepsilon _{j}\big )^{\kappa }\big (\varepsilon _{j'}\big )^{\kappa }\Big )}\Big | \mathrm {P_{j}}\mathrm {P_{j'}}\bigg )\bigg \rangle \\ {}&= \bigg \langle (\rho _{i},s_{ij}), h_{{\mathbb {I}}}\bigg (\big (\phi _{j}\big )^{\kappa }e^{2\pi i\big (\varepsilon _{j}\big )^{\kappa }}\Big | \mathrm {P_{j}}\bigg )\bigg \rangle \otimes \bigg \langle (\rho _{i},s_{ij}), h_{{\mathbb {I}}}\bigg (\big (\phi _{j'}\big )^{\kappa } e^{2\pi i\big (\varepsilon _{j'}\big )^{\kappa }} \Big | \mathrm {P_{j'}}\bigg )\bigg \rangle \\ {}&= {\mathbb {I}}(b_{j})^{\kappa } \otimes {\mathbb {I}}(b_{j'})^{\kappa }\\ {}&= R.H.S. \end{aligned}$$

Thus L.H.S = R.H.S

Hence $$\Big ({\mathbb {I}}(b_{j}) \otimes {\mathbb {I}}(b_{j'})\Big )^{\kappa } = {\mathbb {I}}(b_{j})^{\kappa } \otimes {\mathbb {I}}(b_{j'})^{\kappa }$$ $$\square$$.

(7) $$\kappa _{1}{\mathbb {I}}(b_{j}) \oplus \kappa _{2}{\mathbb {I}}(b_{j}) = ( \kappa _{1} + \kappa _{2}){\mathbb {I}}(b_{j})$$

#### Proof

  L.H.S:

$$\begin{aligned} \kappa _{1} {\mathbb {I}}(b_{j}) \oplus \kappa _{2} {\mathbb {I}}(b_{j}) =&\bigg \langle (\rho _{i},s_{ij}),h_{{\mathbb {I}}}\bigg (\Big (1-\big (1 - \phi _{j}\big )^{\kappa _{1}} \Big ) e^{2\pi i\Big ( 1 - \big (1 - \varepsilon _{j}\big )^{\kappa _{1}}\Big )}\Big | \mathrm {P_{j}}\bigg )\bigg \rangle \oplus \\&\bigg \langle (\rho _{i},s_{ij}),h_{{\mathbb {I}}}\bigg (\Big (1-\big (1 - \phi _{j}\big )^{\kappa _{2}} \Big ) e^{2\pi i\Big ( 1 - \big (1 - \varepsilon _{j}\big )^{\kappa _{2}}\Big )}\Big | \mathrm {P_{j}}\bigg )\bigg \rangle . \end{aligned}$$

Firstly we will consider only amplitude term:

$$\begin{aligned} \Big (1-\big (1 - \phi _{j}\big )^{\kappa _{1}}\Big ) \oplus \Big (1-\big (1 - \phi _{j}\big )^{\kappa _{2}}\Big )&= \Big (1 - \big (1 - \phi _{j}\big )^{\kappa _{1}}\Big ) + \Big (1-\big (1 - \phi _{j}\big )^{\kappa _{2}}\Big ) - \Big (1-\big (1 - \phi _{j}\big )^{\kappa _{1}}\Big )\\ {}&~~~~~\Big (1-\big (1 - \phi _{j}\big )^{\kappa _{2}}\Big )\\ {}&= 2 - \big (1 - \phi _{j}\big )^{\kappa _{1}} -\big (1 - \phi _{j}\big )^{\kappa _{2}} - \Big [ 1 - \big (1 - \phi _{j}\big )^{\kappa _{2}} - \big (1 - \phi _{j}\big )^{\kappa _{1}} +\\ {}&~~~~~ \big (1 - \phi _{j}\big )^{\kappa _{1} + \kappa _{2}} \Big ]\\ {}&= 2 - \big (1 - \phi _{j}\big )^{\kappa _{1}} -\big (1 - \phi _{j}\big )^{\kappa _{2}} - 1 + \big (1 - \phi _{j}\big )^{\kappa _{2}} + \big (1 - \phi _{j}\big )^{\kappa _{1}} - \\ {}&~~~~~\big (1 - \phi _{j}\big )^{\kappa _{1} + \kappa _{2}}\\ {}&= 1 - \big (1 - \phi _{j}\big )^{\kappa _{1} + \kappa _{2}}. \end{aligned}$$

Similarly for phase and probability terms;

$$\begin{aligned} \Big (1-\big (1 - \varepsilon _{j}\big )^{\kappa _{1}}\Big ) \oplus \Big (1-\big (1 - \varepsilon _{j}\big )^{\kappa _{2}}\Big ) = 1 - \big (1 - \varepsilon _{j}\big )^{\kappa _{1} + \kappa _{2}}. \end{aligned}$$

This implies that:

$$\begin{aligned} \kappa _{1} {\mathbb {I}}(b_{j}) \oplus \kappa _{2} {\mathbb {I}}(b_{j})&= \bigg \langle (\rho _{i},s_{ij}),h_{{\mathbb {I}}}\bigg (\Big (1 - \big (1 - \phi _{j}\big )^{\kappa _{1} + \kappa _{2}} \Big ) e^{2\pi i\big (1 - \left(1 - \varepsilon _{j}\right)^{\kappa _{1} + \kappa _{2}}\big )}\big |\mathrm {P_{j}}\bigg )\bigg \rangle \\ {}&= ( \kappa _{1} + \kappa _{2}){\mathbb {I}}(b_{j})\\ {}&= R.H.S. \end{aligned}$$

Thus L.H.S = R.H.S

Hence $$\kappa _{1}{\mathbb {I}}(b_{j}) \oplus \kappa _{2}{\mathbb {I}}(b_{j}) = ( \kappa _{1} + \kappa _{2}){\mathbb {I}}(b_{j})$$. $$\square$$

(8) $${\mathbb {I}}(b_{j})^{\kappa _{1}} \otimes {\mathbb {I}}(b_{j})^{\kappa _{2}} = {\mathbb {I}}(b_{j})^{ \kappa _{1} + \kappa _{2}}$$

#### Proof

L.H.S:

$$\begin{aligned} {\mathbb {I}}(b_{j})^{\kappa _{1}} \otimes {\mathbb {I}}(b_{j'})^{ \kappa _{2}}&= \bigg \langle (\rho _{i},s_{ij}), h_{{\mathbb {I}}}\Big ((\phi _{j})^{\kappa _{1}}e^{2\pi i(\varepsilon _{j})^{\kappa _{1}}}\big | \mathrm {P_{j}}\Big )\bigg \rangle \otimes \bigg \langle (\rho _{i},s_{ij}), h_{{\mathbb {I}}}\Big ((\phi _{j})^{\kappa _{2}}e^{2\pi i(\varepsilon _{j})^{\kappa _{2}}}\big | \mathrm {P_{j}}\Big )\bigg \rangle \\ {}&= \bigg \langle (\rho _{i},s_{ij}), h_{{\mathbb {I}}}\Big (\left((\phi _{j})^{\kappa _{1}}(\phi _{j})^{\kappa _{2}}\right)e^{2\pi i\left((\varepsilon _{j})^{\kappa _{1}}(\varepsilon _{j})^{\kappa _{2}}\right)}\big |\mathrm {P_{j}}\Big )\bigg \rangle \\ {}&=\bigg \langle (\rho _{i},s_{ij}), h_{{\mathbb {I}}}\Big ((\phi _{j})^{\kappa _{1}+\kappa _{2} }e^{2\pi i(\varepsilon _{j})^{\kappa _{1} + \kappa _{2}}}\big | \mathrm {P_{j}}\Big )\bigg \rangle \\ {}&= {\mathbb {I}}(b_{j})^{ \kappa _{1} + \kappa _{2}}\\ {}&= R.H.S. \end{aligned}$$

Thus L.H.S = R.H.S

Hence $${\mathbb {I}}(b_{j})^{\kappa _{1}} \otimes {\mathbb {I}}(b_{j})^{\kappa _{2}} = {\mathbb {I}}(b_{j})^{ \kappa _{1} + \kappa _{2}}$$. $$\square$$

## Aggregation operators for CPHFNSS

In this section we will develop aggregation operators for CPHFNSSs such as CPHFNS weighted averaging aggregation (CPHFNSWA) operator, generalized weighted averaging aggregation (GCPHFNSWA) operator, weighted geometric aggregation (CPHFNSWG) operator, and generalized weighted geometric aggregation (GCPHFNSWG) operator. We will also provide their theorems and related properties such as idempotency, boundedness and monotonicity etc. with proofs.

### Definition 4.1

Let ${\mathbb {I}}(b_{j}) = {\langle } (\rho _{i}, s_{ij}),h_{{\mathbb {I}}}\big (\phi _{j}e^{2\pi i\varepsilon _{j}}{|}\mathrm {P_{j}}\big ){\rangle }$ is the element of CPHFNSS, and let $\upsilon = \{\upsilon _{1}, \upsilon _{2}, \upsilon _{3}, ..., \upsilon _{s}\}$ represents the weight vector of ${\mathbb {I}}(b_{j})$ $(j = 1, 2, 3, ..., s),$ where $\upsilon _{j} \ge 0$ and $\sum \limits _{j = 1}^{s} \upsilon _{j} = 1$. Then the CPHFNSWA operator is defined as:

$$\begin{aligned} CPHFNSWA \left( {\mathbb {I}}(b_{1}), {\mathbb {I}}(b_{2}),..., {\mathbb {I}}(b_{s})\right) = \bigoplus \limits _{j = 1}^{s} \upsilon _{j}{\mathbb {I}}(b_{j}). \end{aligned}$$

### Theorem 4.2

Let ${\mathbb {I}}(b_{j}) = {\langle } (\rho _{i}, s_{ij}),h_{{\mathbb {I}}}\big (\phi _{j}e^{2\pi i\varepsilon _{j}}{|}\mathrm {P_{j}}\big ){\rangle }$ is the element of CPHFNSS, and let $\upsilon = \{\upsilon _{1}, \upsilon _{2}, \upsilon _{3}, ..., \upsilon _{s}\}$ represents the weight vector of ${\mathbb {I}}(b_{j})$ $(j = 1, 2, 3, ..., s),$ where $\upsilon _{j} \ge 0$ and $\sum \limits _{j = 1}^{s} \upsilon _{j} = 1$. Then the CPHFNSWA operator is defined as:

3 $$\begin{aligned} CPHFNSWA&\big ( {\mathbb {I}}(b_{1}), {\mathbb {I}}(b_{2}),..., {\mathbb {I}}(b_{s})\big ) = \bigoplus \limits _{j = 1}^{s} \upsilon _{j}{\mathbb {I}}(b_{j})\\&= \Bigg \langle \big (\rho _{i}, max_{j=1}^{s} s_{ij}\big ), h_{{\mathbb {I}}}\Bigg (\Big ( 1 - \prod \limits _{j = 1}^{s} \left(1 - \phi _{j}\right)^{\upsilon _{j}}\Big ) e^{2\pi i\big ( 1 - \prod \limits _{j = 1}^{s} \left(1 - \varepsilon _{j}\right)^{\upsilon _{j}}\big )}\Big | \prod \limits _{j = 1}^{s}\mathrm {P_{j}}\Bigg )\Bigg \rangle . \end{aligned}$$

### Proof

We will use mathematical induction to prove on s.

$\bullet$ For s = 2, we have

$$\begin{aligned} CPHFNSWA \left({\mathbb {I}}(b_{1}), {\mathbb {I}}(b_{2})\right) = \bigoplus \limits _{j = 1}^{2} \upsilon _{j}{\mathbb {I}}(b_{j}) = \upsilon _{1}{\mathbb {I}}(b_{1})\oplus \upsilon _{2}{\mathbb {I}}(b_{2}). \end{aligned}$$

By using operational laws, we have

$$\begin{aligned} \upsilon _{1}{\mathbb {I}}(b_{1})\oplus \upsilon _{2}{\mathbb {I}}(b_{2}) =&\left\langle (\rho _{i}, s_{i1}),h_{{\mathbb {I}}}\left(\left( 1- \left(1 - \phi _{1}\right)^{\upsilon _{1}}\right)e^{2\pi i\left( 1- \left(1 - \varepsilon _{1}\right)^{\upsilon _{1}}\right)} \big | \mathrm {P_{1}}\right)\right\rangle \oplus \\&\left\langle (\rho _{i}, s_{i2}),h_{{\mathbb {I}}}\left(\left( 1- \left(1 - \phi _{2}\right)^{\upsilon _{2}}\right) e^{2\pi i\left( 1- \left(1 - \varepsilon _{2}\right)^{\upsilon _{2}}\right)} \big | \mathrm {P_{2}}\right)\right\rangle . \end{aligned}$$

We will do firstly for amplitude term;

$$\begin{aligned} 1- \big (1 - \phi _{1}\big )^{\upsilon _{1}} \oplus 1- \big (1 - \phi _{2}\big )^{\upsilon _{2}}&= 1- \big (1 - \phi _{1}\big )^{\upsilon _{1}} + 1- \big (1 - \phi _{2}\big )^{\upsilon _{2}} - \Big (1- \big (1 - \phi _{1}\big )^{\upsilon _{1}}\Big )\Big ( 1- \big (1 - \phi _{2}\big )^{\upsilon _{2}}\Big ) \\ {}&= 1 - \Big (1- \big (1 - \phi _{1}\big )^{\upsilon _{1}}\Big )\Big ( 1- \big (1 - \phi _{2}\big )^{\upsilon _{2}}\Big ). \end{aligned}$$

Similarly for phase term;

$$\begin{aligned} 1- \big (1 - \varepsilon _{1}\big )^{\upsilon _{1}} \oplus 1- \big (1 - \varepsilon _{2}\big )^{\upsilon _{2}} = 1 - \Big (1- \big (1 - \varepsilon _{1}\big )^{\upsilon _{1}}\Big )\Big ( 1- \big (1 - \varepsilon _{2}\big )^{\upsilon _{2}}\Big ). \end{aligned}$$

Thus,

$$\begin{aligned} \upsilon _{1}{\mathbb {I}}(b_{1})\oplus \upsilon _{2}{\mathbb {I}}(b_{2}) = \bigg \langle&\big (\rho _{i}, max_{j=1}^{2} s_{ij}\big ) ,h_{{\mathbb {I}}}\Big (\left(1 - \left(1- \left(1 - \phi _{1}\right)^{\upsilon _{1}}\Big )\Big ( 1- \left(1 - \phi _{2}\right)^{\upsilon _{2}}\right)\right)\\&e^{2\pi i\left( 1 - \left(1- \left(1 - \varepsilon _{1}\right)^{\upsilon _{1}}\right)\left( 1- \left(1 - \varepsilon _{2}\right)^{\upsilon _{2}}\right)\right)} \big | \mathrm {P_{1}}\mathrm {P_{2}}\Big )\bigg \rangle . \end{aligned}$$

$$\Rightarrow$$

It is true for $s = 2$.

$$\bullet$$

Suppose Eq. (3) holds for s = t, that is;

$$\begin{aligned} CPHFNSWA&\left( {\mathbb {I}}(b_{1}), {\mathbb {I}}(b_{2}),..., {\mathbb {I}}(b_{t})\right) = \bigoplus \limits _{j = 1}^{t} \upsilon _{j}{\mathbb {I}}(b_{j}) \\&= \left\langle \big (\rho _{i}, max_{j=1}^{t} s_{ij}\big ) ,h_{{\mathbb {I}}}\Bigg (\Big ( 1 - \prod \limits _{j = 1}^{t} (1 - \phi _{j})^{\upsilon _{j}}\Big )e^{2\pi i( 1 - \prod \limits _{j = 1}^{t} (1 - \varepsilon _{j})^{\upsilon _{j}})}\Big |\prod \limits _{j = 1}^{t} \mathrm {P_{j}}\Bigg )\right\rangle . \end{aligned}$$

Consequently, the overall sum is a CPHFNS number. Continuing that,when s = t +1 , then by using operational laws, we have

$$\begin{aligned} CPHFNSWA&\big ( {\mathbb {I}}(b_{1}), {\mathbb {I}}(b_{2}),..., {\mathbb {I}}(b_{t + 1})\big ) = \bigoplus \limits _{j = 1}^{t + 1} \upsilon _{j}{\mathbb {I}}(b_{j})\\ {}&= \upsilon _{1}{\mathbb {I}}(b_{1})\oplus \upsilon _{2}{\mathbb {I}}(b_{2})\oplus ... \oplus \upsilon _{t}{\mathbb {I}}(b_{t})\oplus \upsilon _{t + 1}{\mathbb {I}}(b_{t + 1})\\&= \left\langle \big (\rho _{i}, max_{j=1}^{t} s_{ij}\big ),h_{{\mathbb {I}}}\Bigg (\bigg ( 1 - \prod \limits _{j = 1}^{t} \left(1 - \phi _{j}\right)^{\upsilon _{j}} \bigg )e^{2\pi i\big ( 1 - \prod \limits _{j = 1}^{t} (1 - \varepsilon _{j})^{\upsilon _{j}}\big )}\big |\prod \limits _{j = 1}^{t} \mathrm {P_{j}}\Bigg )\right\rangle \\&~~~~~ \oplus \left\langle (\rho _{i}, s_{it+1}),h_{{\mathbb {I}}}\left(\left( 1 - \left(1 - \varnothing _{{t+1}}\right)^{\upsilon _{t + 1}} \right) e^{2\pi i\left( 1 - \left(1 - \circleddash _{t+1}\right)^{\upsilon _{t +1}}\right)}\big |\mathrm {P_{t+1}} \right)\right\rangle \\&= \left\langle \big (\rho _{i}, max_{j=1}^{t+1} s_{ij}\big ),h_{{\mathbb {I}}}\Bigg (\bigg ( 1 - \prod \limits _{j = 1}^{t+1} \left(1 - \phi _{j}\right)^{\upsilon _{j}} \bigg )e^{2\pi i\big ( 1 - \prod \limits _{j = 1}^{t+1} (1 - \varepsilon _{j})^{\upsilon _{j}}\big )}\big |\prod \limits _{j = 1}^{t+1} \mathrm {P_{j}}\Bigg )\right\rangle . \end{aligned}$$

Now, Eq. (3) is true for s = t + 1, thus it is true for all s. The proof is completed. $\square$

### Property 4.3

(Idempotency) If  ${\mathbb {I}}(b_{j})$ $(j = 1, 2,..., s)$ all are equal, i.e., ${\mathbb {I}}(b_{j}) = {\mathbb {I}}(b)$ for all j , then

$$\begin{aligned} CPHFSNWA \big ( {\mathbb {I}}(b_{1}), {\mathbb {I}}(b_{2}),..., {\mathbb {I}}(b_{s})\big ) = {\mathbb {I}}(b). \end{aligned}$$

### Proof

Let ${\mathbb {I}}(b_{j}) = {\mathbb {I}}(b)$, and then

$$\begin{aligned} CPHFNSWA \big ( {\mathbb {I}}(b_{1}), {\mathbb {I}}(b_{2}),..., {\mathbb {I}}(b_{s})\big )&= CPHFNSWA \big ( {\mathbb {I}}(b), {\mathbb {I}}(b),..., {\mathbb {I}}(b)\big ) = \bigoplus \limits _{j = 1}^{s} \upsilon _{j}{\mathbb {I}}(b)\\&= \bigg \langle \big (\rho _{i}, max_{j=1}^{s} s_{ij}\big ),h_{{\mathbb {I}}}\bigg (\Big ( 1 - \prod \limits _{j = 1}^{s} (1 - \phi )^{\upsilon _{k}}\Big )e^{2\pi i( 1 - \prod \limits _{j = 1}^{s} (1 - \varepsilon )^{\upsilon _{j}})}\Big | \text{P} \bigg )\bigg \rangle \\&=\bigg \langle (\rho _{i}, s_{ij}),h_{{\mathbb {I}}}\bigg (\Big ( 1 - \big (1 - \phi \big )^{\upsilon _{1}+...+\upsilon _{s}}\Big ) e^{2\pi i\big ( 1 - (1 - \varepsilon )^{\upsilon _{1} +\cdots +\upsilon _{s}}\big )}\Big |\text{P} \bigg )\bigg \rangle . \end{aligned}$$

Since $$\sum \limits _{j = 1}^{s} \upsilon _{j} = 1$$.

$$\begin{aligned} CPHFNSWA \big ( {\mathbb {I}}(b_{1}), {\mathbb {I}}(b_{2}),..., {\mathbb {I}}(b_{s})\big )&= \bigg \langle (\rho _{i}, s_{ij}),h_{{\mathbb {I}}}\bigg (\Big ( 1 -1 + \phi \big ) e^{2\pi i\big ( 1 -1 + \varepsilon \big )}\Big | \text{P}\bigg )\bigg \rangle \\&= \bigg \langle (\rho _{i}, s_{ij}),h_{{\mathbb {I}}}\Big ( \phi ~ e^{2\pi i( \varepsilon )}\Big | \text{P}\Big )\bigg \rangle \\&= {\mathbb {I}}(b). \end{aligned}$$

$$\square$$

### Property 4.4

(Monotonicity) Let ${\mathbb {I}}(b_{j})$ $(j = 1, 2,..., s)$ and ${\mathbb {I}}'(b_{j})$ be the two families of CPHFNSNs. If the grading and hesitant elements of ${\mathbb {I}}(b_{j})\ge {\mathbb {I}}'(b_{j})$, then

$$\begin{aligned} CPHFNSWA \big ( {\mathbb {I}}(j_{1}), {\mathbb {I}}(j_{2}),..., {\mathbb {I}}(j_{s})\big ) \ge CPHFNSWA \big ( {\mathbb {I}}'(j_{1}), {\mathbb {I}}'(j_{2}),..., {\mathbb {I}}'(j_{s})\big ). \end{aligned}$$

### Proof

Because $s_{ij}$ of ${\mathbb {I}}(b_{j})\ge s_{ij}$ of ${\mathbb {I}}'(b_{j})$ and $h_{{\mathbb {I}}} \ge h_{{\mathbb {I}}'}$ we could assume that $\phi _{j} \ge \phi '_{j}$, $\varepsilon _{j} \ge \varepsilon '_{j}$ for all *j*, while sum of probability of each hesitant element is equal to 1. As the collective outcome of CPHFNSWA operator is also an CPHFNSE. We will discuss the amplitude, phase and probability term separately.

Firstly, amplitude term:

As

$$\begin{aligned} \phi _{j}&\ge \phi '_{j}\\ \Rightarrow 1-\phi _{j}&\le 1-\phi '_{j}\\ \prod \limits _{j=1}^{s}( 1-\phi _{j})^{\upsilon _{j}}&\le \prod \limits _{j=1}^{s}(1-\phi '_{j})^{\upsilon _{j}}\\ \Rightarrow 1-\prod \limits _{j=1}^{s}( 1-\phi _{j})^{\upsilon _{j}}&\ge 1- \prod \limits _{j=1}^{s}(1-\phi '_{j})^{\upsilon _{j}}. \end{aligned}$$

Similarly for phase term:

$$\begin{aligned} 1-\prod \limits _{j=1}^{s}( 1-\varepsilon _{j})^{\upsilon _{j}}&\ge 1- \prod \limits _{j=1}^{s}(1-\varepsilon _{j'})^{\upsilon _{j}}. \end{aligned}$$

Also the sum of probability of hesitant element is equal to 1. Thus,

$$\begin{aligned} CPHFNSWA \big ( {\mathbb {I}}(j_{1}), {\mathbb {I}}(j_{2}),..., {\mathbb {I}}(j_{s})\big ) \ge CPHFNSWA \big ( {\mathbb {I}}'(j_{1}), {\mathbb {I}}'(j_{2}),..., {\mathbb {I}}'(j_{s})\big ). \end{aligned}$$

$$\square$$

### Property 4.5

(Boundedness) Let ${\mathbb {I}}(b_{j})$ $(j = 1, 2,..., s)$ be the two family of CPHFNS numbers. The CPHFNSWA lies in between the maximum and minimum operators.

$$\begin{aligned} min\big ( {\mathbb {I}}(b_{1}), {\mathbb {I}}(b_{2}),..., {\mathbb {I}}(b_{s})\big ) \le CPHFNSWA \big ( {\mathbb {I}}(b_{1}), {\mathbb {I}}(b_{2}),..., {\mathbb {I}}(b_{s})\big )\le max\big ( {\mathbb {I}}(b_{1}), {\mathbb {I}}(b_{2}),..., {\mathbb {I}}(b_{s})\big ). \end{aligned}$$

### Proof

Let $$min \big ( {\mathbb {I}}(b_{1}), {\mathbb {I}}(b_{2}),..., {\mathbb {I}}(b_{s})\big )= b$$ and $$max \big ( {\mathbb {I}}(b_{1}), {\mathbb {I}}(b_{2}),..., {\mathbb {I}}(b_{s})\big )= B$$.

Since $b\le CPHFNSWA \big ( {\mathbb {I}}(b_{1}), {\mathbb {I}}(b_{2}),..., {\mathbb {I}}(b_{s})\big ) \le B$. We get the following results by using Property 4.4.

$$\begin{aligned} \bigoplus \limits _{j = 1}^{s} \upsilon _{j}b\le \bigoplus \limits _{j= 1}^{s} \upsilon _{j}{\mathbb {I}}(b_{j}) \le \bigoplus \limits _{j = 1}^{s} \upsilon _{j}B. \end{aligned}$$

This implies that:

$$\begin{aligned} b\le \bigoplus \limits _{j = 1}^{s} \upsilon _{j}{\mathbb {I}}(b_{j}) \le B. \end{aligned}$$

That is:

$$\begin{aligned} min\big ( {\mathbb {I}}(b_{1}), {\mathbb {I}}(b_{2}),..., {\mathbb {I}}(b_{s})\big ) \le CPHFSHA \big ( {\mathbb {I}}(b_{1}), {\mathbb {I}}(b_{2}),..., {\mathbb {I}}(b_{s})\big )\le max\big ( {\mathbb {I}}(b_{1}), {\mathbb {I}}(b_{2}),..., {\mathbb {I}}(b_{s})\big ). \end{aligned}$$

$$\square$$

### Definition 4.6

Let ${\mathbb {I}}(b_{j}) = {\langle } (\rho _{i}, s_{ij}),h_{{\mathbb {I}}}\big (\phi _{j}e^{2\pi i\varepsilon _{j}}{|}\mathrm {P_{j}}\big ){\rangle }$ is the element of CPHFNSS, and let $\upsilon = \{\upsilon _{1}, \upsilon _{2}, \upsilon _{3}, ..., \upsilon _{s}\}$ represents the weight vector of ${\mathbb {I}}(b_{j})$ $(j = 1, 2, 3, ..., s),$ where $\upsilon _{j} \ge 0$ and $\sum \nolimits _{j = 1}^{s} \upsilon _{j} = 1$. Then the GCPHFNSWA operator is defined as:

$$\begin{aligned} GCPHFNSWA \left( {\mathbb {I}}(b_{1}), {\mathbb {I}}(b_{2}),..., {\mathbb {I}}(b_{s})\right) = \Big (\bigoplus \limits _{j = 1}^{s} \upsilon _{j}{\mathbb {I}}(b_{j})^{\kappa }\Big )^{\frac{1}{\kappa }}. \end{aligned}$$

### Theorem 4.7

Let ${\mathbb {I}}(b_{j}) = {\langle } (\rho _{i}, s_{ij}),h_{{\mathbb {I}}}\big (\phi _{j}e^{2\pi i\varepsilon _{j}}{|}\mathrm {P_{j}}\big ){\rangle }$ is the element of CPHFNSS, and let $\upsilon = \{\upsilon _{1}, \upsilon _{2}, \upsilon _{3}, ..., \upsilon _{s}\}$ represents the weight vector of ${\mathbb {I}}(b_{j})$ $(j = 1, 2, 3, ..., s),$ where $\upsilon _{j} \ge 0$ and $\sum \limits _{j = 1}^{s} \upsilon _{j} = 1$. Then the GCPHFNSWA operator is defined as:

$$\begin{aligned} GCPHFNSWA&\big ( {\mathbb {I}}(b_{1}), {\mathbb {I}}(b_{2}),..., {\mathbb {I}}(b_{s})\big ) = \Big (\bigoplus \limits _{j = 1}^{s} \upsilon _{j}{\mathbb {I}}(b_{j})^{\kappa }\Big )^{\frac{1}{\kappa }}\\&= \Bigg \langle \big (\rho _{i}, max_{j=1}^{s} s_{ij}\big ), h_{{\mathbb {I}}}\Bigg (\Big ( 1 - \prod \limits _{j = 1}^{s} \big (1 - \phi _{j}^{\kappa }\big )^{\upsilon _{j}}\Big )^{\frac{1}{\kappa }} e^{2\pi i(\Big ( 1 - \prod \limits _{j = 1}^{s} \big (1 - \varepsilon _{j}^{\kappa }\big )^{\upsilon _{j}}\Big )^{\frac{1}{\kappa }}}\Big | \prod \limits _{j = 1}^{s}\mathrm {P_{j}}\Bigg )\Bigg \rangle . \end{aligned}$$

The proof can be demonstrated analogously as above proof so we omit it.

### Property 4.8

(Idempotency) If  ${\mathbb {I}}(b_{j})$ $(j = 1, 2,..., s)$ all are equal, i.e., ${\mathbb {I}}(b_{j}) = {\mathbb {I}}(b)$ for all j , then

$$\begin{aligned} GCPHFSNWA \big ( {\mathbb {I}}(b_{1}), {\mathbb {I}}(b_{2}),..., {\mathbb {I}}(b_{s})\big ) = {\mathbb {I}}(b). \end{aligned}$$

### Property 4.9

(Monotonicity) Let ${\mathbb {I}}(b_{j})$ $(j = 1, 2,..., s)$ and ${\mathbb {I}}'(b_{j})$ be the two families of CPHFNS numbers. If the grading and hesitant elements of ${\mathbb {I}}(b_{j})\ge {\mathbb {I}}'(b_{j})$, then

$$\begin{aligned} GCPHFNSWA \big ( {\mathbb {I}}(j_{1}), {\mathbb {I}}(j_{2}),..., {\mathbb {I}}(j_{s})\big ) \ge GCPHFNSWA \big ( {\mathbb {I}}'(j_{1}), {\mathbb {I}}'(j_{2}),..., {\mathbb {I}}'(j_{s})\big ). \end{aligned}$$

### Property 4.10

(Boundedness) Let ${\mathbb {I}}(b_{j})$ $(j = 1, 2,..., s)$ be the two family of CPHFNS numbers. The GCPHFNSWA lies in between the maximum and minimum operators.

$$\begin{aligned} min\big ( {\mathbb {I}}(b_{1}), {\mathbb {I}}(b_{2}),..., {\mathbb {I}}(b_{s})\big ) \le GCPHFNSWA \big ( {\mathbb {I}}(b_{1}), {\mathbb {I}}(b_{2}),..., {\mathbb {I}}(b_{s})\big )\le max\big ( {\mathbb {I}}(b_{1}), {\mathbb {I}}(b_{2}),..., {\mathbb {I}}(b_{s})\big ). \end{aligned}$$

### Definition 4.11

Let ${\mathbb {I}}(b_{j}) = {\langle } (\rho _{i}, s_{ij}),h_{{\mathbb {I}}}\big (\phi _{j}e^{2\pi i\varepsilon _{j}}{|}\mathrm {P_{j}}\big ){\rangle }$ is the element of CPHFNSS, and let $\upsilon = \{\upsilon _{1}, \upsilon _{2}, \upsilon _{3}, ..., \upsilon _{s}\}$ represents the weight vector of ${\mathbb {I}}(b_{j})$ $(j = 1, 2, 3, ..., s),$ where $\upsilon _{j} \ge 0$ and $\sum \limits _{j = 1}^{s} \upsilon _{j} = 1$. Then the CPHFNSWG operator is defined as:

$$\begin{aligned} CPHFNSWG \left( {\mathbb {I}}(b_{1}), {\mathbb {I}}(b_{2}),..., {\mathbb {I}}(b_{s})\right) = \bigotimes \limits _{j = 1}^{s} ({\mathbb {I}}(b_{j}))^{\upsilon _{j}}. \end{aligned}$$

### Theorem 4.12

Let ${\mathbb {I}}(b_{j}) = {\langle } (\rho _{i}, s_{ij}),h_{{\mathbb {I}}}\big (\phi _{j}e^{2\pi i\varepsilon _{j}}{|}\mathrm {P_{j}}\big ){\rangle }$ is the element of CPHFNSS, and let $\upsilon = \{\upsilon _{1}, \upsilon _{2}, \upsilon _{3}, ..., \upsilon _{s}\}$ represents the weight vector of ${\mathbb {I}}(b_{j})$ $(j = 1, 2, 3, ..., s),$ where $\upsilon _{j} \ge 0$ and $\sum \limits _{j = 1}^{s} \upsilon _{j} = 1$. Then the CPHFNSWG operator is defined as:

4 $$\begin{aligned} CPHFNSWG&\big ( {\mathbb {I}}(b_{1}), {\mathbb {I}}(b_{2}),..., {\mathbb {I}}(b_{s})\big ) = \bigotimes \limits _{j = 1}^{s} {\mathbb {I}}(b_{j})\upsilon _{j}\\&= \Bigg \langle \big (\rho _{i}, min_{j=1}^{s} s_{ij}\big ), h_{{\mathbb {I}}}\Bigg (\Big ( \prod \limits _{j = 1}^{s} \phi _{j}^{\upsilon _{j}}\Big ) e^{2\pi i\big ( \prod \limits _{j = 1}^{s} \varepsilon _{j}^{\upsilon _{j}}\big )}\Big | \prod \limits _{j = 1}^{s}\mathrm {P_{j}}\Bigg )\Bigg \rangle . \end{aligned}$$

### Proof

We will use mathematical induction to prove on s.

$\bullet$ For s = 2, we have

$$\begin{aligned} CPHFNSWG \left({\mathbb {I}}(b_{1}), {\mathbb {I}}(b_{2})\right) = \bigotimes \limits _{j = 1}^{2} {\mathbb {I}}(b_{j})^{\upsilon _{j}} = {\mathbb {I}}(b_{1})^{\upsilon _{1}}\otimes {\mathbb {I}}(b_{2})^{\upsilon _{2}}. \end{aligned}$$

By using operational laws, we have

$$\begin{aligned} {\mathbb {I}}(b_{1})^{\upsilon _{1}}\otimes {\mathbb {I}}(b_{2})^{\upsilon _{2}}&= \left\langle (\rho _{i}, s_{i1}),h_{{\mathbb {I}}}\left( \phi _{1}^{\upsilon _{1}}e^{2\pi i\left(\varepsilon _{1}^{\upsilon _{1}}\right)} \big | \mathrm {P_{1}}\right) \right\rangle \otimes \left\langle (\rho _{i}, s_{i2}),h_{{\mathbb {I}}}\left(\phi _{2}^{\upsilon _{2}} e^{2\pi i\left( \varepsilon _{2}^{\upsilon _{2}}\right)} \big | \mathrm {P_{2}}\right)\right\rangle \\&= \bigg \langle \big (\rho _{i}, min_{j=1}^{2} s_{ij}\big ) ,h_{{\mathbb {I}}}\Big (\left(\phi _{1}^{\upsilon _{1}} \phi _{2}^{\upsilon _{2}}\right) e^{2\pi i\left( \varepsilon _{1}^{\upsilon _{1}}\varepsilon _{2}^{\upsilon _{2}}\right)} \big | \mathrm {P_{1}}\mathrm {P_{2}}\Big )\bigg \rangle . \end{aligned}$$

$\Rightarrow$ It is true for $s = 2$.

$\bullet$ Suppose Eq. (4) holds for s = t, that is;

$$\begin{aligned} CPHFNSWG&\left( {\mathbb {I}}(b_{1}), {\mathbb {I}}(b_{2}),..., {\mathbb {I}}(b_{t})\right) = \bigotimes \limits _{j = 1}^{t} {\mathbb {I}}(b_{j})^{\upsilon _{j}} \\&= \left\langle \big (\rho _{i}, min_{j=1}^{t} s_{ij}\big ) ,h_{{\mathbb {I}}}\Bigg (\bigg (\prod \limits _{j = 1}^{t} \phi _{j}^{\upsilon _{j}}\bigg )e^{2\pi i\Big ( \prod \limits _{j = 1}^{t} \varepsilon _{j}^{\upsilon _{j}}\Big )}\Big |\prod \limits _{j = 1}^{t} \mathrm {P_{j}}\Bigg )\right\rangle . \end{aligned}$$

Consequently, the overall sum is a CPHFNS number. Continuing that, when s = t +1 , then by using operational laws, we have

$$\begin{aligned} CPHFNSWA \big ( {\mathbb {I}}(b_{1}), {\mathbb {I}}(b_{2}),..., {\mathbb {I}}(b_{t + 1})\big )&= \bigotimes \limits _{j = 1}^{t + 1} {\mathbb {I}}(b_{j})^{\upsilon _{j}}\\ {}&= {\mathbb {I}}(b_{1})^{\upsilon _{1}}\otimes {\mathbb {I}}(b_{2})\upsilon _{2}\otimes ... \otimes {\mathbb {I}}(b_{t})^{\upsilon _{t}}\otimes {\mathbb {I}}(b_{t + 1})^{\upsilon _{t + 1}}\\&= \left\langle \big (\rho _{i}, min_{j=1}^{t} s_{ij}\big ),h_{{\mathbb {I}}}\Bigg (\bigg ( \prod \limits _{j = 1}^{t} \phi _{j}^{\upsilon _{j}} \bigg )e^{2\pi i\big ( \prod \limits _{j = 1}^{t} \varepsilon _{j}^{\upsilon _{j}}\big )}\big |\prod \limits _{j = 1}^{t} \mathrm {P_{j}}\Bigg )\right\rangle \\&~~~~~ \otimes \left\langle (\rho _{i}, s_{it+1}),h_{{\mathbb {I}}}\left(\left(\varnothing _{{t+1}}^{\upsilon _{t + 1}} \right) e^{2\pi i\left(\circleddash _{t+1}^{\upsilon _{t +1}}\right)}\big |\mathrm {P_{t+1}} \right)\right\rangle \\&= \left\langle \big (\rho _{i}, min_{j=1}^{t+1} s_{ij}\big ),h_{{\mathbb {I}}}\Bigg (\bigg (\prod \limits _{j = 1}^{t+1}\phi _{j}^{\upsilon _{j}} \bigg )e^{2\pi i\big ( \prod \limits _{j = 1}^{t+1}\varepsilon _{j}^{\upsilon _{j}}\big )}\big |\prod \limits _{j = 1}^{t+1} \mathrm {P_{j}}\Bigg )\right\rangle . \end{aligned}$$

Now, Eq. (4) is true for s = t + 1, thus it is true for all s. The proof is completed. $\square$

### Property 4.13

(Idempotency) If  ${\mathbb {I}}(b_{j})$ $(j = 1, 2,..., s)$ all are equal, i.e., ${\mathbb {I}}(b_{j}) = {\mathbb {I}}(b)$ for all j , then

$$\begin{aligned} CPHFSNWG \big ( {\mathbb {I}}(b_{1}), {\mathbb {I}}(b_{2}),..., {\mathbb {I}}(b_{s})\big ) = {\mathbb {I}}(b). \end{aligned}$$

### Proof

Let ${\mathbb {I}}(b_{j}) = {\mathbb {I}}(b)$, and then

$$\begin{aligned} CPHFNSWG \big ( {\mathbb {I}}(b_{1}), {\mathbb {I}}(b_{2}),..., {\mathbb {I}}(b_{s})\big )&= CPHFNSWG \big ( {\mathbb {I}}(b), {\mathbb {I}}(b),..., {\mathbb {I}}(b)\big ) = \bigotimes \limits _{j = 1}^{s} {\mathbb {I}}(b)^{\upsilon _{j}}\\&= \bigg \langle \big (\rho _{i}, min_{j=1}^{s} s_{ij}\big ),h_{{\mathbb {I}}}\bigg (\Big ( \prod \limits _{j = 1}^{s}\phi ^{\upsilon _{k}}\Big )e^{2\pi i( \prod \limits _{j = 1}^{s} \varepsilon ^{\upsilon _{j}})}\Big | \text{P} \bigg )\bigg \rangle \\&=\bigg \langle (\rho _{i}, s_{ij}),h_{{\mathbb {I}}}\bigg (\Big ( \phi ^{\upsilon _{1}+...+\upsilon _{s}}\Big ) e^{2\pi i\big ( \varepsilon ^{\upsilon _{1} +...+\upsilon _{s}}\big )}\Big |\text{P} \bigg )\bigg \rangle . \end{aligned}$$

Since $$\sum \nolimits _{j = 1}^{s} \upsilon _{j} = 1$$.

$$\begin{aligned} CPHFNSWG \big ( {\mathbb {I}}(b_{1}), {\mathbb {I}}(b_{2}),..., {\mathbb {I}}(b_{s})\big )&= \bigg \langle (\rho _{i}, s_{ij}),h_{{\mathbb {I}}}\bigg (\Big ( \phi ~ e^{2\pi i\big ( \varepsilon \big )}\Big | \text{P}\bigg )\bigg \rangle \\&= \bigg \langle (\rho _{i}, s_{ij}),h_{{\mathbb {I}}}\Big ( \phi ~ e^{2\pi i( \varepsilon )}\Big | \text{P}\Big )\bigg \rangle \\&= {\mathbb {I}}(b). \end{aligned}$$

$$\square$$

### Property 4.14

(Monotonicity) Let ${\mathbb {I}}(b_{j})$ $(j = 1, 2,..., s)$ and ${\mathbb {I}}'(b_{j})$ be the two families of CPHFNS numbers. If the grading and hesitant elements of ${\mathbb {I}}(b_{j})\ge {\mathbb {I}}'(b_{j})$, then

$$\begin{aligned} CPHFNSWG \big ( {\mathbb {I}}(j_{1}), {\mathbb {I}}(j_{2}),..., {\mathbb {I}}(j_{s})\big ) \ge CPHFNSWG \big ( {\mathbb {I}}'(j_{1}), {\mathbb {I}}'(j_{2}),..., {\mathbb {I}}'(j_{s})\big ). \end{aligned}$$

### Proof

Because $s_{ij}$ of ${\mathbb {I}}(b_{j})\ge s_{ij}$ of ${\mathbb {I}}'(b_{j})$ and $h_{{\mathbb {I}}} \ge h_{{\mathbb {I}}'}$ we could assume that $\phi _{j} \ge \phi '_{j}$, $\varepsilon _{j} \ge \varepsilon '_{j}$ for all *j*, while sum of probability of each hesitant element is equal to 1. As the collective outcome of CPHFNSWG operator is also an CPHFNSE. We will discuss the amplitude, phase and probability term separately.

Firstly, amplitude term:

As

$$\begin{aligned} \phi _{j}&\ge \phi '_{j}\\ \prod \limits _{j=1}^{s}( \phi _{j})^{\upsilon _{j}}&\ge \prod \limits _{j=1}^{s}(\phi '_{j})^{\upsilon _{j}}. \end{aligned}$$

Similarly for phase term:

$$\begin{aligned} \prod \limits _{j=1}^{s}(\varepsilon _{j})^{\upsilon _{j}}&\ge \prod \limits _{j=1}^{s}(\varepsilon _{j'})^{\upsilon _{j}}. \end{aligned}$$

Also the sum of probability of hesitant element is equal to 1. Thus,

$$\begin{aligned} CPHFNSWG \big ( {\mathbb {I}}(j_{1}), {\mathbb {I}}(j_{2}),..., {\mathbb {I}}(j_{s})\big ) \ge CPHFNSWG \big ( {\mathbb {I}}'(j_{1}), {\mathbb {I}}'(j_{2}),..., {\mathbb {I}}'(j_{s})\big ). \end{aligned}$$

$$\square$$

### Property 4.15

(Boundedness) Let ${\mathbb {I}}(b_{j})$ $(j = 1, 2,..., s)$ be the two family of CPHFNS numbers. The CPHFNSWG lies in between the maximum and minimum operators.

$$\begin{aligned} min\big ( {\mathbb {I}}(b_{1}), {\mathbb {I}}(b_{2}),..., {\mathbb {I}}(b_{s})\big ) \le CPHFNSWG \big ( {\mathbb {I}}(b_{1}), {\mathbb {I}}(b_{2}),..., {\mathbb {I}}(b_{s})\big )\le max\big ( {\mathbb {I}}(b_{1}), {\mathbb {I}}(b_{2}),..., {\mathbb {I}}(b_{s})\big ). \end{aligned}$$

### Proof

Let $$min \big ( {\mathbb {I}}(b_{1}), {\mathbb {I}}(b_{2}),..., {\mathbb {I}}(b_{s})\big )= b$$ and $$max \big ( {\mathbb {I}}(b_{1}), {\mathbb {I}}(b_{2}),..., {\mathbb {I}}(b_{s})\big )= B$$.

Since $b'\le CPHFNSWG \big ( {\mathbb {I}}(b_{1}), {\mathbb {I}}(b_{2}),..., {\mathbb {I}}(b_{s})\big ) \le B'$. We get the following results by using Property 4.14.

$$\begin{aligned} \bigotimes \limits _{j = 1}^{s} b'^{\upsilon _{j}}\le \bigotimes \limits _{j= 1}^{s} {\mathbb {I}}(b_{j})^{\upsilon _{j}} \le \bigotimes \limits _{j = 1}^{s} B'^{\upsilon _{j}}. \end{aligned}$$

This implies that:

$$\begin{aligned} b'\le \bigotimes \limits _{j = 1}^{s} {\mathbb {I}}(b_{j})^{\upsilon _{j}} \le B'. \end{aligned}$$

That is:

$$\begin{aligned} min\big ( {\mathbb {I}}(b_{1}), {\mathbb {I}}(b_{2}),..., {\mathbb {I}}(b_{s})\big ) \le CPHFSHG \big ( {\mathbb {I}}(b_{1}), {\mathbb {I}}(b_{2}),..., {\mathbb {I}}(b_{s})\big )\le max\big ( {\mathbb {I}}(b_{1}), {\mathbb {I}}(b_{2}),..., {\mathbb {I}}(b_{s})\big ). \end{aligned}$$

$$\square$$

### Definition 4.16

Let ${\mathbb {I}}(b_{j}) = {\langle } (\rho _{i}, s_{ij}),h_{{\mathbb {I}}}\big (\phi _{j}e^{2\pi i\varepsilon _{j}}{|}\mathrm {P_{j}}\big ){\rangle }$ is the element of CPHFNSS, and let $\upsilon = \{\upsilon _{1}, \upsilon _{2}, \upsilon _{3}, ..., \upsilon _{s}\}$ represents the weight vector of ${\mathbb {I}}(b_{j})$ $(j = 1, 2, 3, ..., s),$ where $\upsilon _{j} \ge 0$ and $\sum \limits _{j = 1}^{s} \upsilon _{j} = 1$. Then the GCPHFNSWG operator is defined as:

$$\begin{aligned} GCPHFNSWG \left( {\mathbb {I}}(b_{1}), {\mathbb {I}}(b_{2}),..., {\mathbb {I}}(b_{s})\right) = \frac{1}{\kappa }\bigotimes \limits _{j = 1}^{s}(\kappa ({\mathbb {I}}(b_{j}))^{\upsilon _{j}}. \end{aligned}$$

### Theorem 4.17

Let ${\mathbb {I}}(b_{j}) = {\langle } (\rho _{i}, s_{ij}),h_{{\mathbb {I}}}\big (\phi _{j}e^{2\pi i\varepsilon _{j}}{|}\mathrm {P_{j}}\big ){\rangle }$ is the element of CPHFNSS, and let $\upsilon = \{\upsilon _{1}, \upsilon _{2}, \upsilon _{3}, ..., \upsilon _{s}\}$ represents the weight vector of ${\mathbb {I}}(b_{j})$ $(j = 1, 2, 3, ..., s),$ where $\upsilon _{j} \ge 0$ and $\sum \limits _{j = 1}^{s} \upsilon _{j} = 1$. Then the GCPHFNSWG operator is defined as:

$$\begin{aligned} G&CPHFNSWG \big ( {\mathbb {I}}(b_{1}), {\mathbb {I}}(b_{2}),..., {\mathbb {I}}(b_{s})\big ) = \frac{1}{\kappa }\bigotimes \limits _{j = 1}^{s}(\kappa ({\mathbb {I}}(b_{j}))^{\upsilon _{j}}\\&= \Bigg \langle \big (\rho _{i}, min_{j=1}^{s} s_{ij}\big ), h_{{\mathbb {I}}}\Bigg (\Big (1 - \Big ( 1 - \prod \limits _{j = 1}^{s} \big (1 - \big (1 - \phi _{j}\big )^{\kappa }\big )^{\upsilon _{j}}\Big )^{\frac{1}{\kappa }}\Big ) e^{2\pi i(\Big (1 - \Big ( 1 - \prod \limits _{j = 1}^{s} \big (1 - \big (1 - \varepsilon _{j}\big )^{\kappa }\big )^{\upsilon _{j}}\Big )^{\frac{1}{\kappa }}\Big )}\Big | \prod \limits _{j = 1}^{s}\mathrm {P_{j}}\Bigg )\Bigg \rangle . \end{aligned}$$

The proof can be demonstrated analogously as above proof so we omit it.

### Property 4.18

(Idempotency) If  ${\mathbb {I}}(b_{j})$ $(j = 1, 2,..., s)$ all are equal, i.e., ${\mathbb {I}}(b_{j}) = {\mathbb {I}}(b)$ for all j , then

$$\begin{aligned} GCPHFSNWG \big ( {\mathbb {I}}(b_{1}), {\mathbb {I}}(b_{2}),..., {\mathbb {I}}(b_{s})\big ) = {\mathbb {I}}(b). \end{aligned}$$

### Property 4.19

(**Monotonicity**) Let ${\mathbb {I}}(b_{j})$ $(j = 1, 2,..., s)$ and ${\mathbb {I}}'(b_{j})$ be the two families of CPHFNS numbers. If the grading and hesitant elements of ${\mathbb {I}}(b_{j})\ge {\mathbb {I}}'(b_{j})$, then

$$\begin{aligned} GCPHFNSWG \big ( {\mathbb {I}}(j_{1}), {\mathbb {I}}(j_{2}),..., {\mathbb {I}}(j_{s})\big ) \ge GCPHFNSWG \big ( {\mathbb {I}}'(j_{1}), {\mathbb {I}}'(j_{2}),..., {\mathbb {I}}'(j_{s})\big ). \end{aligned}$$

### Property 4.20

(Boundedness) Let ${\mathbb {I}}(b_{j})$ $(j = 1, 2,..., s)$ be the two family of CPHFNS numbers. The GCPHFNSWG lies in between the maximum and minimum operators.

$$\begin{aligned} min\big ( {\mathbb {I}}(b_{1}), {\mathbb {I}}(b_{2}),..., {\mathbb {I}}(b_{s})\big ) \le GCPHFNSWG \big ( {\mathbb {I}}(b_{1}), {\mathbb {I}}(b_{2}),..., {\mathbb {I}}(b_{s})\big )\le max\big ( {\mathbb {I}}(b_{1}), {\mathbb {I}}(b_{2}),..., {\mathbb {I}}(b_{s})\big ). \end{aligned}$$

## Framework of multi-parameter group decision making

In this section, we will put forth the decision-making framework that captures the ambiguity and considers healthcare-challenging situations while also taking into account the psychological state of the decision-makers. Finding the optimal solution to an issue is usually vital for achieving the greatest outcome. Algorithms are the process through which individuals make choices. An algorithm is a predefined, comprehensive sequence of actions that provides the optimal solution to a particular issue. If complete precision is desired, it is better to employ an algorithm since the usage of an algorithm improves accuracy and reduces the risk of mistakes.

In this work, we provide an algorithm for selecting choices using CPHF data that takes into account parameters and grading where we assume *k* distinct alternatives $\mathbb {Z} = \{\rho _{1}, \rho _{2}, \rho _{3},..., \rho _{k} \}$ and *s* parameters $\mathbb {B}= \{b_{1}, b_{2},..., b_{s}\}$ and the weight vectors for each parameter is $\upsilon = \{\upsilon _{1}, \upsilon _{2}, \upsilon _{3}, ..., \upsilon _{s}\}$ where $\upsilon _{j} \ge 0$, $(j = 1, 2, ..., s),$ and $\sum \nolimits _{j = 1}^{s} \upsilon _{j} = 1$. Consider a situation where there are *y* decision-makers, ${\mathcal {X}}= \{x_{1}, x_{2},..., x_{y}\}$ and their assigning weight vectors are $\eta = \{\eta _{1}, \eta _{2}, \eta _{3}, ..., \eta _{y}\}$, where $\eta _{\alpha } \ge 0$, $(\alpha = 1, 2,..., y),$ and $\sum \nolimits _{\alpha = 1}^{y} \eta _{\alpha } = 1$.

**Table:** 

Algorithm 5.1.	
*Step 1* Take as input the universal set, the set of experts, and the set of parameters with their corresponding weight vectors.	
*Step 2* Gather evaluation data from each specialists on the parameters of each alternative, and then construct the CPHFNSS matrix.	
*Step 3* Normalize the CPHFNSS matrix by using the Definition 3.9 if the criteria is cost type. While the benefit type criteria need no further actions.	
*Step 4* Use the GCPHFNSWA or GCPHFNSWG operator to aggregate the information of each parameter relative to each alternative of all decision-makers as follows.	
$${\mathcal {X}}(b_{j}) = GCPHFNSWA \big ( x_{1}(b_{j}), x_{2}(b_{j}),..., x_{y}(b_{j})\big ) = \Big (\bigoplus \limits _{\alpha = 1}^{y} \eta _{\alpha }\big (x_{\alpha }(b_{j})\big )^{\kappa }\Big )^{\frac{1}{\kappa }}$$	
$$\text {or}$$	
$${\mathcal {X}}'(b_{j}) = GCPHFNSWG \big ( x_{1}(b_{j}), x_{2}(b_{j}),..., x_{y}(b_{j})\big ) = \frac{1}{\kappa }\bigotimes \limits _{\alpha = 1}^{y}(\kappa (x_{\alpha }(b_{j}))^{\eta _{\alpha }}$$	
*Step 5* Aggregate the parameters of each alternative, using the CPHFNSWA or CPHFNSWG operator.	
*Step 6* Apply the Definitions 3.14–3.17 to figure out the score for each alternative.	
*Step 7* Rank all viable options in decreasing order, then choose the best desirable choice as the output.	

### A case study in healthcare decision-making

**Figure 1:**
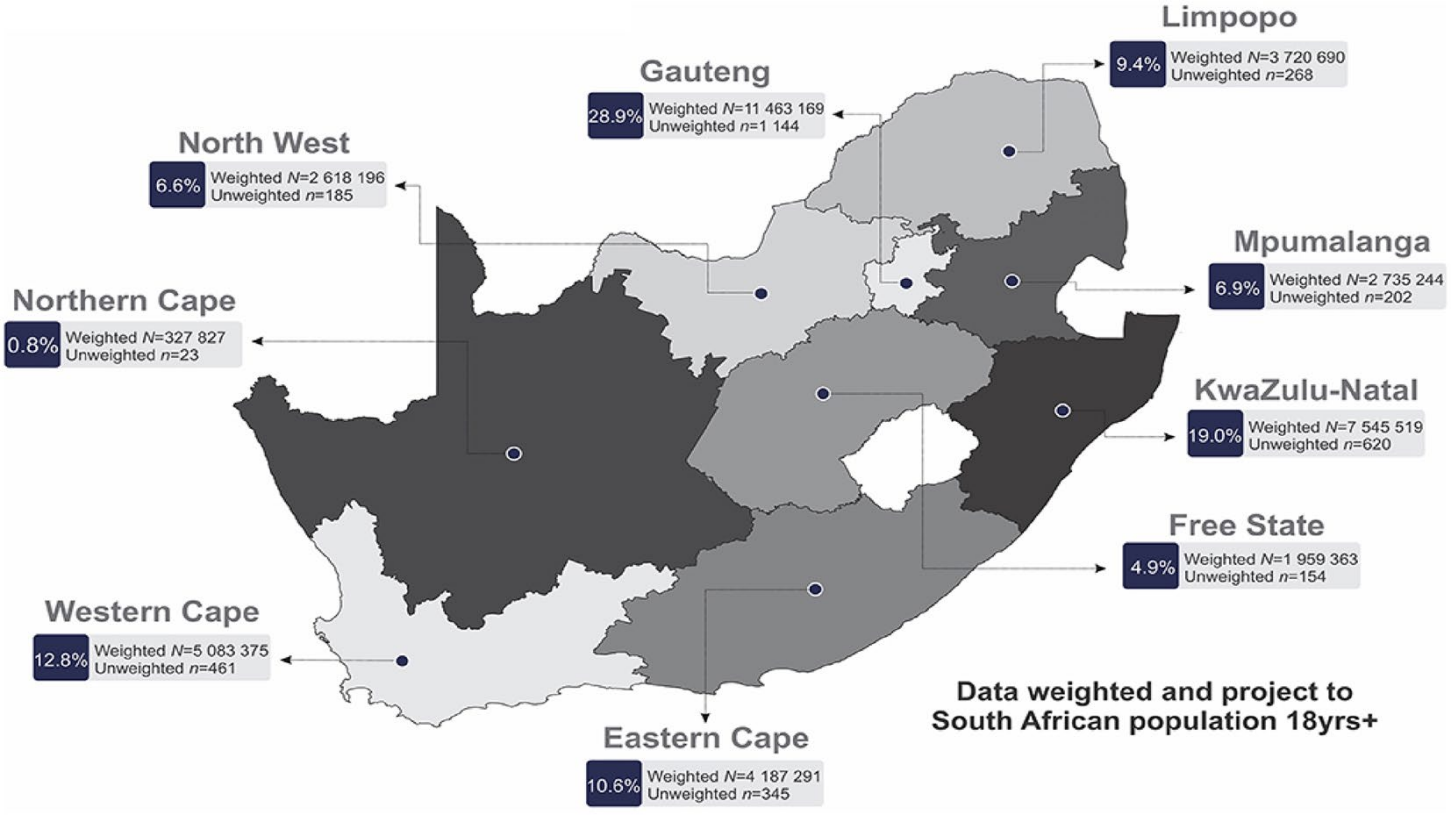
Demographics of the nine provinces of South Africa’s population.

**Figure 2:**
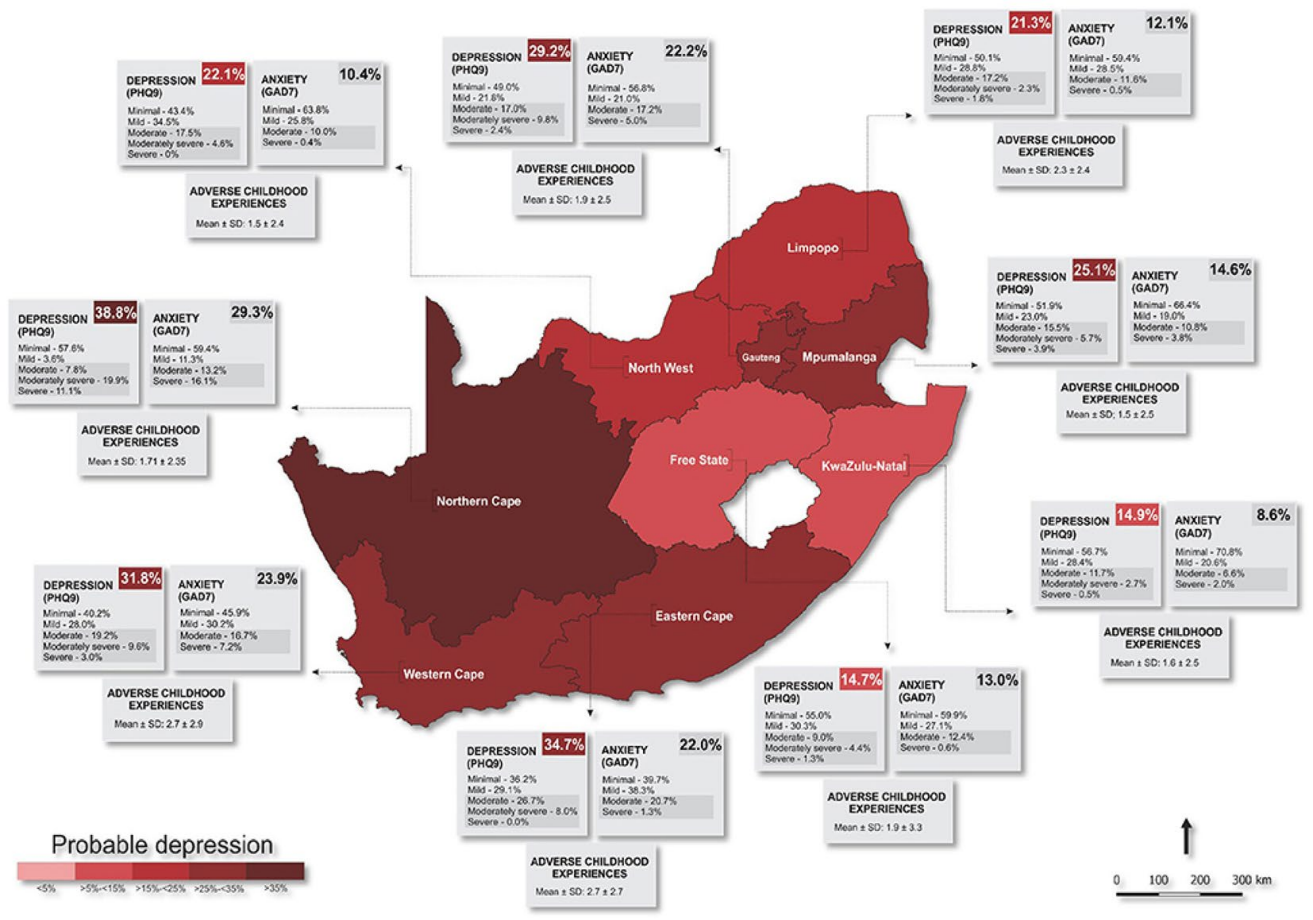
Prevalence of mental health risk across South Africa.

**Figure 3:**
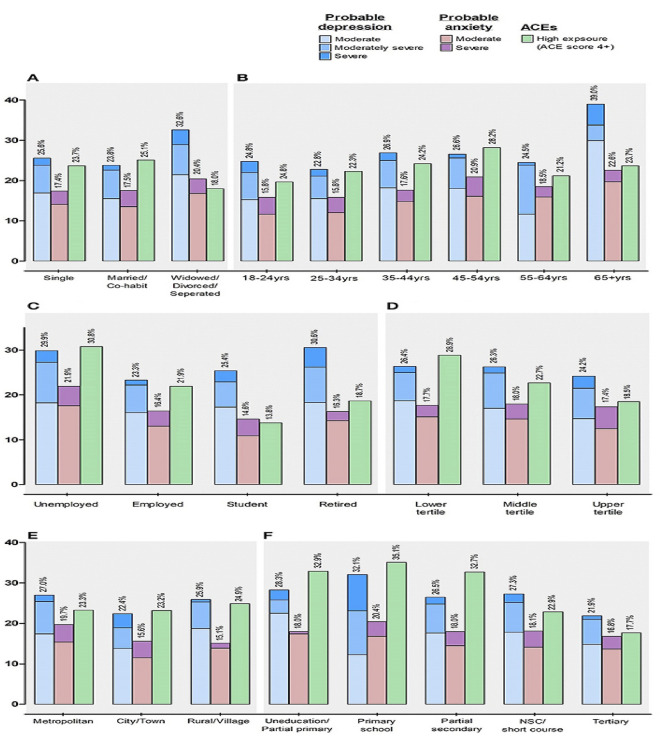
Mental health risk stratified.

The prevalence of mental health issues among people in low- and middle-income nations is a growing public health concern. Mental health issues are widespread in South Africa, with bad childhood experiences, socioeconomic position, geographical region, age, parental status, and levels of education influencing the incidence of mental disorder. The case study is taken from the article “ The prevalence of probable depression and probable anxiety, and associations with adverse childhood experiences and socio-demographics: A national survey in South Africa” published on 28 October in 2022^54^ (Figs. 1, 2, 3).

In a 2009 research conducted in South Africa, a middle-income nation with large economic disparities, it was found that approximately 20% of people suffer from poor mental health, with fewer than 25% of this group ever receiving mental health therapy. According to a 2013 research, the Eastern Cape, one of the poorest regions in South Africa, has the highest lifetime prevalence rate of depression (31.4%). A 2018 research done in urban informal settlements in South Africa indicated that almost one in five women expressed moderate to severe anxiety. It has also been stated that more than 50% of South African adults had been subjected to adverse childhood experiences (ACEs) such as emotional or sexual abuse throughout childhood, with around forty percent having suffered some kind of emotional neglect before the age of eighteen^54^. In 2022 the cross-sectional research examined a nationally representative sample of people (aged 18 and above) by^54^. This research demonstrated that more than one-fourth of South Africans suffer from probable depression, with various regions having greater rates. During the course of nine provinces in South Africa which are graphically represented in Fig. 1 from^54^, a team of 180 experienced fieldworkers drew on a well-established network of research facilities and a streamlined method for coordinating their efforts to gather data. It demonstrates that 17.8% of respondents indicated probable anxiety (GAD-7) and 23.6% reported significant exposure to ACE. The prevalence of probable depression, anxiety, and adverse childhood experiences varied across the country’s nine provinces, according to their findings. The graphical representation of this study illustrated in Fig. 2 from^54^. While the risk factors for mental illness are classified according to marital status by (A); age categories by (B); employment status by (C); household asset score by (D); urban city by (E); and education level by (F) is illustrated in Fig. 3 from^54^.

There are many different mental disorders that exist, and the exact number can vary depending on how they are classified and diagnosed. The most widely used classification system is the Diagnostic and Statistical Manual of Mental Disorders (DSM-5), which identifies and defines more than 200 different mental disorders. Inadequacies in mental health treatment have been linked to a variety of factors. The absence of a comprehensive mental health strategy is a serious infrastructure and planning issue. So, if society wants to offer individuals with better healthcare and increase their level of life, it is essential that we comprehend, treat, and prevent mental disease. Physicians may use Algorithm 1 scores to assess a patient’s overall prognosis, and researchers can use them to identify specific risks faced by people with multiple mental disorders. In this case, we provide an example to help visualize the numerical example.

There exist two doctors for the examination of patients. Let $\mathbb {Z}= \{\rho _{1}, \rho _{2}, \rho _{3}, \rho _{4}, \rho _{5}\}$ be the set of disorders and the set of the parameters consist of symptoms or clinical characteristics involved in these disorders which is under consideration that is, $\mathbb {B}=\{b_{1}, b_{2}, b_{3}\}$. The weight vectors for each parameter is $\upsilon = \{0.25, 0.4, 0.35\}$ where $\upsilon _{j} \ge 0$, $(j = 1, 2, 3),$ and $\sum \limits _{j = 1}^{3} \upsilon _{j} = 1$. $\mathbb {R}= \{0, 1, 2, 3, 4, 5, 6, 7, 8, 9\}$ be the set of ordered grades concerning the severity of disease, where zero represents that there exist no severity of the disease while 9 indicate the entire severity of the disease. The doctors will collect the data in CPHFNSS as described in *Step 2* where the amplitude term of membership grade represents the belongingness of the disease and the phase term represents the durability of the symptom concerning the disease while probability indicates how likely is that a particular statement given by the patient is accurate. Their assigning weight vectors are $\eta = \{0.55, 0.45\}$, where $\eta _{\alpha } \ge 0$, $(\alpha = 1, 2),$ and $\sum \limits _{\alpha = 1}^{2} \eta _{\alpha } = 1$. Tables 4 and 5 represent the data collected by the doctors from the patient.

**Table 4:** Data collected by the doctor 1.

$({x_{1}},\mathbb {B}_{1},10)$	$b_{1}$	$b_{2}$	$b_{3}$
$\rho _{1}$	$$\left( 4, \left\{ \begin{array}{l} 0.7e^{2\pi i 0.7}|0.5,\\ 0.6e^{2\pi i 0.7}|0.5 \end{array} \right\} \right)$$	$$\left( 6, \left\{ \begin{array}{l} 0.3e^{2\pi i 0.8}|0.4,\\ 0.2e^{2\pi i 0.4}|0.6 \end{array} \right\} \right)$$	$$\left( 2, \left\{ \begin{array}{l} 0.3e^{2\pi i 0.9}|1 \end{array} \right\} \right)$$
$\rho _{2}$	$$\left( 9, \left\{ \begin{array}{l} 0.6e^{2\pi i 0.6}|1 \end{array} \right\} \right)$$	$$\left( 0, \left\{ \begin{array}{l} 0.8e^{2\pi i 0.4}|0.1,\\ 0.6e^{2\pi i 0.1}|0.9 \end{array} \right\} \right)$$	$$\left( 8, \left\{ \begin{array}{l} 0.3e^{2\pi i 0.8}|0.3,\\ 0.3e^{2\pi i 0.5}|0.7 \end{array} \right\} \right)$$
$\rho _{3}$	$$\left( 7, \left\{ \begin{array}{l} 0.4e^{2\pi i 0.7}|1 \end{array} \right\} \right)$$	$$\left( 7, \left\{ \begin{array}{l} 0.5e^{2\pi i 0.6}|1 \end{array} \right\} \right)$$	$$\left( 4, \left\{ \begin{array}{l} 0.4e^{2\pi i 0.3}|0.3,\\ 0.5e^{2\pi i 0.8}|0.7 \end{array} \right\} \right)$$
$\rho _{4}$	$$\left( 1, \left\{ \begin{array}{l} 0.2e^{2\pi i 0.9}|0.4,\\ 0.5e^{2\pi i 0.3}|0.6 \end{array} \right\} \right)$$	$$\left( 7, \left\{ \begin{array}{l} 0.3e^{2\pi i 0.8}|1 \end{array} \right\} \right)$$	$$\left( 3, \left\{ \begin{array}{l} 0.2e^{2\pi i 0.1}|1 \end{array} \right\} \right)$$
$\rho _{5}$	$$\left( 3, \left\{ \begin{array}{l} 0.4e^{2\pi i 0.6}|0.2,\\ 0.8e^{2\pi i 0.6}|0.8 \end{array} \right\} \right)$$	$$\left( 5, \left\{ \begin{array}{l} 0.3e^{2\pi i 0.2}|0.1,\\ 0.6e^{2\pi i 0.4}|0.9 \end{array} \right\} \right)$$	$$\left( 1, \left\{ \begin{array}{l} 0.1e^{2\pi i 0.1}|1 \end{array} \right\} \right)$$

**Table 5:** Data collected by the doctor 2.

$({x_{2}},\mathbb {B}_{2},10)$	$b_{1}$	$b_{2}$	$b_{3}$
$\rho _{1}$	$$\left( 3, \left\{ \begin{array}{l} 0.8e^{2\pi i 0.7}|0.5,\\ 0.7e^{2\pi i 0.6}|0.5 \end{array} \right\} \right)$$	$$\left( 7, \left\{ \begin{array}{l} 0.2e^{2\pi i 0.5}|1 \end{array} \right\} \right)$$	$$\left( 2, \left\{ \begin{array}{l} 0.3e^{2\pi i 0.9}|1 \end{array} \right\} \right)$$
$\rho _{2}$	$$\left( 8, \left\{ \begin{array}{l} 0.5e^{2\pi i 0.5}|1 \end{array} \right\} \right)$$	$$\left( 1, \left\{ \begin{array}{l} 0.9e^{2\pi i 0.5}|0.4,\\ 0.8e^{2\pi i 0.3}|0.6 \end{array} \right\} \right)$$	$$\left( 8, \left\{ \begin{array}{l} 0.3e^{2\pi i 0.5}|1 \end{array} \right\} \right)$$
$\rho _{3}$	$$\left( 8, \left\{ \begin{array}{l} 0.5e^{2\pi i 0.7}|1 \end{array} \right\} \right)$$	$$\left( 7, \left\{ \begin{array}{l} 0.5e^{2\pi i 0.6}|1 \end{array} \right\} \right)$$	$$\left( 3, \left\{ \begin{array}{l} 0.5e^{2\pi i 0.6}|0.4,\\ 0.5e^{2\pi i 0.8}|0.6 \end{array} \right\} \right)$$
$\rho _{4}$	$$\left( 0, \left\{ \begin{array}{l} 0.3e^{2\pi i 0.8}|0.4,\\ 0.4e^{2\pi i 0.4}|0.6 \end{array} \right\} \right)$$	$$\left( 8, \left\{ \begin{array}{l} 0.3e^{2\pi i 0.8}|0.4,\\ 0.4e^{2\pi i 0.8}|0.6 \end{array} \right\} \right)$$	$$\left( 2, \left\{ \begin{array}{l} 0.1e^{2\pi i 0.1}|1 \end{array} \right\} \right)$$
$\rho _{5}$	$$\left( 3, \left\{ \begin{array}{l} 0.4e^{2\pi i 0.6}|0.2,\\ 0.8e^{2\pi i 0.6}|0.8 \end{array} \right\} \right)$$	$$\left( 6, \left\{ \begin{array}{l} 0.4e^{2\pi i 0.3}|0.2,\\ 0.6e^{2\pi i 0.5}|0.8 \end{array} \right\} \right)$$	$$\left( 2, \left\{ \begin{array}{l} 0.2e^{2\pi i 0.1}|1 \end{array} \right\} \right)$$

*Step 3* There is no need of normalization.

*Step 4* Here we will aggregate the information of all doctors with respect to each parameter of each alternative as represented in Tables 6 and 7 using GCPHFNSWA and GCPHFNSWG operator respectively and $\kappa = 1$.

**Table 6:** Tabular representation of experts collected data.

$({\mathcal {X}},\mathbb {B},10)$	$b_{1}$	$b_{2}$	$b_{3}$
$\rho _{1}$	$$\left( 4, \left\{ \begin{array}{l} 0.75e^{2\pi i 0.7}|0.25,\\ 0.7e^{2\pi i 0.659}|0.25,\\ 0.707e^{2\pi i 0.7}|0.25,\\ 0.649e^{2\pi i 0.659}|0.25 \end{array} \right\} \right)$$	$$\left( 7, \left\{ \begin{array}{l}0.257e^{2\pi i 0.698}|0.4,\\ 0.2e^{2\pi i 0.447}|0.6 \end{array} \right\} \right)$$	$$\left( 2, \left\{ \begin{array}{l} 0.3e^{2\pi i 0.9}|1 \end{array} \right\} \right)$$
$\rho _{2}$	$$\left( 9, \left\{ \begin{array}{l} 0.558e^{2\pi i 0.558}|1 \end{array} \right\} \right)$$	$$\left( 1, \left\{ \begin{array}{l} 0.854e^{2\pi i 0.447}|0.04,\\ 0.8e^{2\pi i 0.357}|0.06,\\ 0.786e^{2\pi i 0.309}|0.36,\\ 0.707e^{2\pi i 0.196}|0.54 \end{array} \right\} \right)$$	$$\left( 8, \left\{ \begin{array}{l} 0.3e^{2\pi i 0.698}|0.3,\\ 0.3e^{2\pi i 0.5}|0.7 \end{array} \right\} \right)$$
$\rho _{3}$	$$\left( 8, \left\{ \begin{array}{l} 0.447e^{2\pi i 0.7}|1 \end{array} \right\} \right)$$	$$\left( 7, \left\{ \begin{array}{l} 0.5e^{2\pi i 0.6}|1 \end{array} \right\} \right)$$	$$\left( 4, \left\{ \begin{array}{l} 0.447e^{2\pi i 0.456}|0.12,\\ 0.447e^{2\pi i 0.602}|0.18,\\ 0.5e^{2\pi i 0.727}|0.28,\\ 0.5e^{2\pi i 0.8}|0.42 \end{array} \right\} \right)$$
$\rho _{4}$	$$\left( 1, \left\{ \begin{array}{l} 0.247e^{2\pi i 0.863}|0.16,\\ 0.297e^{2\pi i 0.776}|0.24,\\ 0.418e^{2\pi i 0.602}|0.24,\\ 0.457e^{2\pi i 0.347}|0.36 \end{array} \right\} \right)$$	$$\left( 8, \left\{ \begin{array}{l} 0.3e^{2\pi i 0.8}|0.4,\\ 0.347e^{2\pi i 0.8}|0.6 \end{array} \right\} \right)$$	$$\left( 3, \left\{ \begin{array}{l} 0.156e^{2\pi i 0.1}|1 \end{array} \right\} \right)$$
$\rho _{5}$	$$\left( 3, \left\{ \begin{array}{l} 0.4e^{2\pi i 0.6}|0.04,\\ 0.634e^{2\pi i 0.6}|0.16,\\ 0.672e^{2\pi i 0.6}|0.16,\\ 0.8e^{2\pi i 0.6}|0.64 \end{array} \right\} \right)$$	$$\left( 6, \left\{ \begin{array}{l} 0.347e^{2\pi i 0.247}|0.02,\\ 0.456e^{2\pi i 0.353}|0.08,\\ 0.52e^{2\pi i 0.357}|0.18,\\ 0.6e^{2\pi i 0.447}|0.72 \end{array} \right\} \right)$$	$$\left( 2, \left\{ \begin{array}{l} 0.146e^{2\pi i 0.1}|1 \end{array} \right\} \right)$$

**Table 7:** Tabular representation of experts collected data.

$({\mathcal {X}}',\mathbb {B},10)$	$b_{1}$	$b_{2}$	$b_{3}$
$\rho _{1}$	$$\left( 3, \left\{ \begin{array}{l} 0.743e^{2\pi i 0.7}|0.25,\\ 0.7e^{2\pi i 0.653}|0.25,\\ 0.683e^{2\pi i 0.7}|0.25,\\ 0.643e^{2\pi i 0.653}|0.25 \end{array} \right\} \right)$$	$$\left( 6, \left\{ \begin{array}{l} 0.25e^{2\pi i 0.647}|0.4,\\ 0.2e^{2\pi i 0.442}|0.6 \end{array} \right\} \right)$$	$$\left( 2, \left\{ \begin{array}{l} 0.3e^{2\pi i 0.9}|1 \end{array} \right\} \right)$$
$\rho _{2}$	$$\left( 8, \left\{ \begin{array}{l} 0.553e^{2\pi i 0.553}|1 \end{array} \right\} \right)$$	$$\left( 0, \left\{ \begin{array}{l} 0.844e^{2\pi i 0.442}|0.04,\\ 0.8e^{2\pi i 0.351}|0.06,\\ 0.72e^{2\pi i 0.206}|0.36,\\ 0.683e^{2\pi i 0.164}|0.54 \end{array} \right\} \right)$$	$$\left( 8, \left\{ \begin{array}{l} 0.3e^{2\pi i 0.647}|0.3,\\ 0.3e^{2\pi i 0.5}|0.7 \end{array} \right\} \right)$$
$\rho _{3}$	$$\left( 7, \left\{ \begin{array}{l} 0.442e^{2\pi i 0.7}|1 \end{array} \right\} \right)$$	$$\left( 7, \left\{ \begin{array}{l} 0.5e^{2\pi i 0.6}|1 \end{array} \right\} \right)$$	$$\left( 3, \left\{ \begin{array}{l} 0.442e^{2\pi i 0.41}|0.12,\\ 0.442e^{2\pi i 0.466}|0.18,\\ 0.5e^{2\pi i 0.703}|0.28,\\ 0.5e^{2\pi i 0.8}|0.42 \end{array} \right\} \right)$$
$\rho _{4}$	$$\left( 0, \left\{ \begin{array}{l} 0.24e^{2\pi i 0.854}|0.16,\\ 0.273e^{2\pi i 0.625}|0.24,\\ 0.397e^{2\pi i 0.466}|0.24,\\ 0.452e^{2\pi i 0.341}|0.36 \end{array} \right\} \right)$$	$$\left( 7, \left\{ \begin{array}{l} 0.3e^{2\pi i 0.8}|0.4,\\ 0.341e^{2\pi i 0.8}|0.6 \end{array} \right\} \right)$$	$$\left( 2, \left\{ \begin{array}{l} 0.146e^{2\pi i 0.1}|1 \end{array} \right\} \right)$$
$\rho _{5}$	$$\left( 3, \left\{ \begin{array}{l} 0.4e^{2\pi i 0.6}|0.04,\\ 0.546e^{2\pi i 0.6}|0.16,\\ 0.586e^{2\pi i 0.6}|0.16,\\ 0.8e^{2\pi i 0.6}|0.64 \end{array} \right\} \right)$$	$$\left( 5, \left\{ \begin{array}{l} 0.341e^{2\pi i 0.24}|0.02,\\ 0.41e^{2\pi i 0.302}|0.08,\\ 0.5e^{2\pi i 0.351}|0.18,\\ 0.6e^{2\pi i 0.442}|0.72 \end{array} \right\} \right)$$	$$\left( 1, \left\{ \begin{array}{l} 0.137e^{2\pi i 0.1}|1 \end{array} \right\} \right)$$

*Step 5* Aggregate the parameters of each alternative using $\upsilon$ weight vectors as represented in Table 8.

**Table 8:** Parametric aggregation.

Alternatives	*CPHFNSWA*	*CPHFNSWG*
$\rho _{1}$	$$\left( 7, \left\{ \begin{array}{l} 0.4457e^{2\pi i 0.7952}|0.1,\\ 0.4198e^{2\pi i 0.7885}|0.1,\\ 0.4234e^{2\pi i 0.7952}|0.1,\\ 0.3964e^{2\pi i 0.7885}|0.1\\ 0.4292e^{2\pi i 0.7392}|0.15,\\ 0.4026e^{2\pi i 0.7306}|0.15,\\ 0.4062e^{2\pi i 0.7392}|0.15,\\ 0.3784e^{2\pi i 0.7306}|0.15 \end{array} \right\} \right)$$	$$\left( 2, \left\{ \begin{array}{l} 0.3499e^{2\pi i 0.7409}|0.1,\\ 0.32e^{2\pi i 0.6361}|0.1,\\ 0.3447e^{2\pi i 0.7282}|0.1,\\ 0.3153e^{2\pi i 0.6252}|0.1\\ 0.3426e^{2\pi i 0.7409}|0.15,\\ 0.3133e^{2\pi i 0.6361}|0.15,\\ 0.3375e^{2\pi i 0.7282}|0.15,\\ 0.3087e^{2\pi i 0.6252}|0.15 \end{array} \right\} \right)$$
$\rho _{2}$	$$\left( 9, \left\{ \begin{array}{l} 0.6662e^{2\pi i 0.5769}|0.012,\\ 0.6219e^{2\pi i 0.5505}|0.018,\\ 0.6113e^{2\pi i 0.5374}|0.108,\\ 0.5596e^{2\pi i 0.5085}|0.162,\\ 0.6662e^{2\pi i 0.4953}|0.028,\\ 0.6219e^{2\pi i 0.4637}|0.042,\\ 0.6113e^{2\pi i 0.4482}|0.252,\\ 0.5596e^{2\pi i 0.4137}|0.378 \end{array} \right\} \right)$$	$$\left( 1, \left\{ \begin{array}{l} 0.5285e^{2\pi i 0.5344}|0.012,\\ 0.5285e^{2\pi i 0.4881}|0.018,\\ 0.5174e^{2\pi i 0.4874}|0.108,\\ 0.5174e^{2\pi i 0.4453}|0.162\\ 0.4961e^{2\pi i 0.3939}|0.028,\\ 0.4961e^{2\pi i 0.3598}|0.042,\\ 0.4857e^{2\pi i 0.3593}|0.252,\\ 0.4857e^{2\pi i 0.4402}|0.378 \end{array} \right\} \right)$$
$\rho _{3}$	$$\left( 8, \left\{ \begin{array}{l} 0.469e^{2\pi i 0.5954}|0.12,\\ 0.469e^{2\pi i 0.6283}|0.18,\\ 0.4873e^{2\pi i 0.6743}|0.28,\\ 0.4873e^{2\pi i 0.7079}|0.42 \end{array} \right\} \right)$$	$$\left( 4, \left\{ \begin{array}{l} 0.4645e^{2\pi i 0.5457}|0.12,\\ 0.4645e^{2\pi i 0.571}|0.18,\\ 0.4849e^{2\pi i 0.6591}|0.28,\\ 0.4849e^{2\pi i 0.6896}|0.42 \end{array} \right\} \right)$$
$\rho _{4}$	$$\left( 8, \left\{ \begin{array}{l} 0.2389e^{2\pi i 0.6922}|0.064,\\ 0.252e^{2\pi i 0.6517}|0.096,\\ 0.2866e^{2\pi i 0.5978}|0.096,\\ 0.2988e^{2\pi i 0.5449}|0.144,\\ 0.2598e^{2\pi i 0.6922}|0.096,\\ 0.2725e^{2\pi i 0.6517}|0.144,\\ 0.3061e^{2\pi i 0.5978}|0.144,\\ 0.318e^{2\pi i 0.5449}|0.216 \end{array} \right\} \right)$$	$$\left( 1, \left\{ \begin{array}{l} 0.2207e^{2\pi i 0.3927}|0.064,\\ 0.2325e^{2\pi i 0.3927}|0.096,\\ 0.228e^{2\pi i 0.3632}|0.096,\\ 0.2401e^{2\pi i 0.3632}|0.144,\\ 0.2504e^{2\pi i 0.3376}|0.096,\\ 0.2637e^{2\pi i 0.3376}|0.144,\\ 0.2586e^{2\pi i 0.3123}|0.144,\\ 0.2723e^{2\pi i 0.3123}|0.216 \end{array} \right\} \right)$$
$\rho _{5}$	$$\left( 6, \left\{ \begin{array}{l} 0.2978e^{2\pi i 0.3156}|0.0008,\\ 0.3794e^{2\pi i 0.3156}|0.0032,\\ 0.3963e^{2\pi i 0.3156}|0.0072,\\ 0.4665e^{2\pi i 0.3156}|0.0288,\\ 0.3472e^{2\pi i 0.3558}|0.0032,\\ 0.4231e^{2\pi i 0.3558}|0.0128,\\ 0.4388e^{2\pi i 0.3558}|0.0288,\\ 0.504e^{2\pi i 0.3558}|0.1152,\\ 0.3792e^{2\pi i 0.3576}|0.0032,\\ 0.4513e^{2\pi i 0.3576}|0.0128,\\ 0.4662e^{2\pi i 0.3576}|0.0288,\\ 0.5283e^{2\pi i 0.3576}|0.1152,\\ 0.4228e^{2\pi i 0.3953}|0.0128,\\ 0.4899e^{2\pi i 0.3953}|0.0512,\\ 0.5038e^{2\pi i 0.3953}|0.1152,\\ 0.5615e^{2\pi i 0.3953}|0.4608 \end{array} \right\} \right)$$	$$\left( 2, \left\{ \begin{array}{l} 0.2578e^{2\pi i 0.2221}|0.0008,\\ 0.2773e^{2\pi i 0.2435}|0.0032,\\ 0.3003e^{2\pi i 0.2587}|0.0072,\\ 0.323e^{2\pi i 0.2837}|0.0288,\\ 0.2787e^{2\pi i 0.2221}|0.0032,\\ 0.2998e^{2\pi i 0.2435}|0.0128,\\ 0.3246e^{2\pi i 0.2587}|0.0288,\\ 0.3492e^{2\pi i 0.2837}|0.1152,\\ 0.2836e^{2\pi i 0.2221}|0.0032,\\ 0.305e^{2\pi i 0.2435}|0.0128,\\ 0.3303e^{2\pi i 0.2587}|0.0288,\\ 0.3553e^{2\pi i 0.2837}|0.1152,\\ 0.3066e^{2\pi i 0.2221}|0.0128,\\ 0.3298e^{2\pi i 0.2435}|0.0512,\\ 0.3571e^{2\pi i 0.2587}|0.1152,\\ 0.3841e^{2\pi i 0.2837}|0.4608 \end{array} \right\} \right)$$

*Step 6* Evaluation of score values of each alternative is represented in Table 9.

**Table 9:** Parametric aggregation.

Alternatives	*CPHFNSWA*	*CPHFNSWG*
$\rho _{1}$	0.05681	0.01404
$\rho _{2}$	0.06538	0.00638
$\rho _{3}$	0.1279	0.06229
$\rho _{4}$	0.04965	0.00413
$\rho _{5}$	0.01882	0.00442

*Step 7* Rank all viable options in decreasing order, then choose the best desirable choice as the output as presented in Table 10.

**Table 10:** Ranking results.

*CPHFNSWA*	*CPHFNSWG*
$\rho _{3}> \rho _{2}> \rho _{1}> \rho _{4} > \rho _{5}$	$\rho _{3}> \rho _{1}> \rho _{2}> \rho _{5} > \rho _{4}$

**Figure 4:**
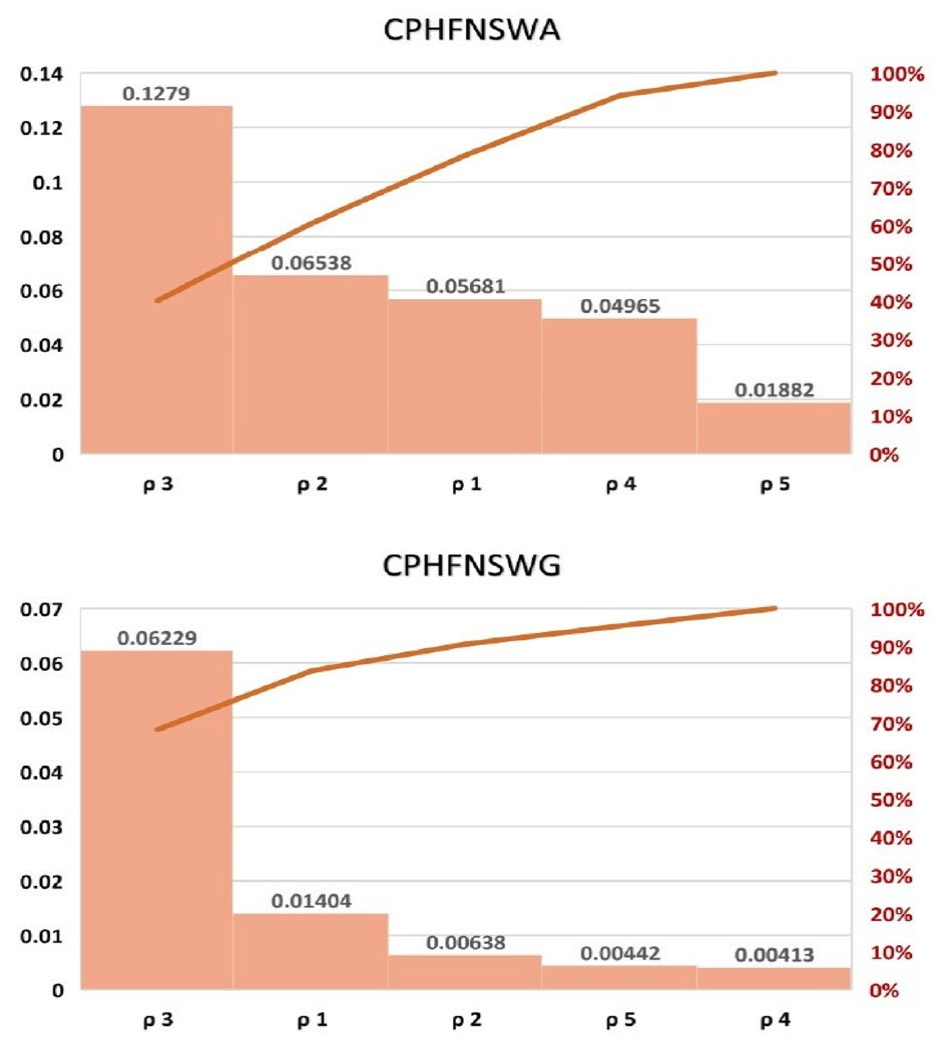
Ranking results.

This indicates that the patient has greater chances to suffer with $\rho _{3}$ mental disorder as compared to others. The graphical representation of the ranking is illustrated in Fig. 4.

The choice between averaging and geometric aggregation operators depends on the specific context and the goals of the aggregation process. Here are some general guidelines for when each operator might be preferred:

Averaging aggregation:

- When the input values are on the same scale and have similar magnitudes.
- When the input values are subject to measurement errors or other sources of variability that can be smoothed out by taking the average.

Geometric aggregation:

- When the input values are on different scales or have different magnitudes and need to be normalized or standardized.
- When the goal is to obtain an aggregate value that reflects the combined effect of multiple input values, and that is more sensitive to small changes in the input values.

## Comparison analysis

We devised an approach by combining the theory of constraints with multi-parameter and multiobjective decision making techniques in order to find a solution to a challenging problem that still exists in the real world. In the context of the CPHFNSS, we examined two different algorithms. Both approaches provide a ranked list of all viable options, regardless of whether or not there is a single best answer. However, none of the existing works are up to the task of handling the kind of information supplied by CPHFNSS to a decision maker. While the suggested method is capable of handling data from pre-existing methods as well, it has the potential to significantly improve upon them. Several structures supplied by various researchers were compared to show how well the proposed method performed in contrast to the existing methods for multi-parameter group decision-making. The comparison is given in Table 11, which concludes that our proposed study is superior and more reliable than the ones currently in use.

**Table 11:** Comparison with existing approaches.

Methods	Capable of making decisions using probability	Capable of handling two dimensional information	Flexible to adapt decision-makers’ choices	Capable of integrate information	Flexible to adapt gradings
Zadeh^1^	No	No	No	No	No
Torra^2^	No	No	Yes	No	No
Akram et al.^40^	No	No	Yes	No	Yes
Akram et al.^55^	No	No	Yes	Yes	Yes
Garg et al.^56^	No	Yes	Yes	No	No
Zhang et al.^57^	Yes	No	Yes	Yes	No
Mahmood et al.^58^	No	Yes	No	No	Yes
Proposed approach	Yes	Yes	Yes	Yes	Yes

Further, we compare our proposed method with the other methods. This method consist of the soft max-AND operator introduced by Ashraf et al.^53^. The group data is represented in the Tables 12 and 13 while the comparison results are available in Table 14.

**Table 12:** Data collected by the expert 1.

$({A_{1}},\mathbb {B}_{1},10)$	$b_{1}$	$b_{2}$	$b_{3}$
$\rho _{1}$	$$\left( 4, \left\{ \begin{array}{l} 0.71e^{2\pi i 0.72}|0.5,\\ 0.63e^{2\pi i 0.72}|0.5 \end{array} \right\} \right)$$	$$\left( 6, \left\{ \begin{array}{l} 0.33e^{2\pi i 0.83}|0.4,\\ 0.22e^{2\pi i 0.44}|0.6 \end{array} \right\} \right)$$	$$\left( 2, \left\{ \begin{array}{l} 0.33e^{2\pi i 0.92}|1 \end{array} \right\} \right)$$
$\rho _{2}$	$$\left( 9, \left\{ \begin{array}{l} 0.63e^{2\pi i 0.63}|1 \end{array} \right\} \right)$$	$$\left( 0, \left\{ \begin{array}{l} 0.81e^{2\pi i 0.44}|0.1,\\ 0.63e^{2\pi i 0.12}|0.9 \end{array} \right\} \right)$$	$$\left( 8, \left\{ \begin{array}{l} 0.33e^{2\pi i 0.84}|0.3,\\ 0.33e^{2\pi i 0.52}|0.7 \end{array} \right\} \right)$$
$\rho _{3}$	$$\left( 7, \left\{ \begin{array}{l} 0.44e^{2\pi i 0.72}|1 \end{array} \right\} \right)$$	$$\left( 7, \left\{ \begin{array}{l} 0.53e^{2\pi i 0.61}|1 \end{array} \right\} \right)$$	$$\left( 4, \left\{ \begin{array}{l} 0.43e^{2\pi i 0.33}|0.3,\\ 0.53e^{2\pi i 0.81}|0.7 \end{array} \right\} \right)$$
$\rho _{4}$	$$\left( 1, \left\{ \begin{array}{l} 0.21e^{2\pi i 0.92}|0.4,\\ 0.52e^{2\pi i 0.33}|0.6 \end{array} \right\} \right)$$	$$\left( 7, \left\{ \begin{array}{l} 0.33e^{2\pi i 0.82}|1 \end{array} \right\} \right)$$	$$\left( 3, \left\{ \begin{array}{l} 0.22e^{2\pi i 0.13}|1 \end{array} \right\} \right)$$
$\rho _{5}$	$$\left( 3, \left\{ \begin{array}{l} 0.42e^{2\pi i 0.63}|0.2,\\ 0.84e^{2\pi i 0.63}|0.8 \end{array} \right\} \right)$$	$$\left( 5, \left\{ \begin{array}{l} 0.32e^{2\pi i 0.24}|0.1,\\ 0.63e^{2\pi i 0.44}|0.9 \end{array} \right\} \right)$$	$$\left( 1, \left\{ \begin{array}{l} 0.12e^{2\pi i 0.12}|1 \end{array} \right\} \right)$$

**Table 13:** Data collected by the expert 2.

$({A_{2}},\mathbb {B}_{2},10)$	$b_{1}$	$b_{2}$	$b_{3}$
$\rho _{1}$	$$\left( 3, \left\{ \begin{array}{l} 0.81e^{2\pi i 0.72}|0.5,\\ 0.73e^{2\pi i 0.61}|0.5 \end{array} \right\} \right)$$	$$\left( 7, \left\{ \begin{array}{l} 0.23e^{2\pi i 0.54}|1 \end{array} \right\} \right)$$	$$\left( 2, \left\{ \begin{array}{l} 0.32e^{2\pi i 0.93}|1 \end{array} \right\} \right)$$
$\rho _{2}$	$$\left( 8, \left\{ \begin{array}{l} 0.51e^{2\pi i 0.54}|1 \end{array} \right\} \right)$$	$$\left( 1, \left\{ \begin{array}{l} 0.92e^{2\pi i 0.53}|0.4,\\ 0.83e^{2\pi i 0.34}|0.6 \end{array} \right\} \right)$$	$$\left( 8, \left\{ \begin{array}{l} 0.32e^{2\pi i 0.51}|1 \end{array} \right\} \right)$$
$\rho _{3}$	$$\left( 8, \left\{ \begin{array}{l} 0.53e^{2\pi i 0.72}|1 \end{array} \right\} \right)$$	$$\left( 7, \left\{ \begin{array}{l} 0.53e^{2\pi i 0.64}|1 \end{array} \right\} \right)$$	$$\left( 3, \left\{ \begin{array}{l} 0.52e^{2\pi i 0.63}|0.4,\\ 0.54e^{2\pi i 0.81}|0.6 \end{array} \right\} \right)$$
$\rho _{4}$	$$\left( 0, \left\{ \begin{array}{l} 0.32e^{2\pi i 0.83}|0.4,\\ 0.44e^{2\pi i 0.44}|0.6 \end{array} \right\} \right)$$	$$\left( 8, \left\{ \begin{array}{l} 0.32e^{2\pi i 0.83}|0.4,\\ 0.41e^{2\pi i 0.81}|0.6 \end{array} \right\} \right)$$	$$\left( 2, \left\{ \begin{array}{l} 0.14e^{2\pi i 0.12}|1 \end{array} \right\} \right)$$
$\rho _{5}$	$$\left( 3, \left\{ \begin{array}{l} 0.42e^{2\pi i 0.62}|0.2,\\ 0.84e^{2\pi i 0.62}|0.8 \end{array} \right\} \right)$$	$$\left( 6, \left\{ \begin{array}{l} 0.43e^{2\pi i 0.32}|0.2,\\ 0.64e^{2\pi i 0.54}|0.8 \end{array} \right\} \right)$$	$$\left( 2, \left\{ \begin{array}{l} 0.22e^{2\pi i 0.13}|1 \end{array} \right\} \right)$$

**Table 14:** Comparison with other method.

Methods	Ranking
Soft max-AND	$\rho _{3}> \rho _{1}> \rho _{4}> \rho _{2} > \rho _{5}$
CPHFNSWG	$\rho _{3}> \rho _{1}> \rho _{2}> \rho _{5} > \rho _{4}$
CPHFNSWA	$\rho _{3}> \rho _{2}> \rho _{1}> \rho _{4} > \rho _{5}$

Upon careful examination of the table, it becomes evident that $\rho _{3}$ is the most advantageous solution when compared to all other approaches. The observed result provides justification for the validity and applicability of the presented methodology. Furthermore, the ranking results reported in this research differ from those obtained using other methodologies due to the limitations of these methods. Aggregation operators play a crucial role in the integration and manipulation of data across many domains, presenting significant benefits over specialized operators such as the soft max-AND and soft min-OR operators. There are many benefits associated with the use of aggregation operators in a broad context.

- In the proposed methodologies, it is feasible to assign weights to decision makers and parameters based on their respective levels of significance, a capability that was absent in prior approaches based on CPHFNSS.
- Aggregation operators have the capability to provide valuable insights into the entire decision-making process via the assignment of weights or degrees of priority to various aspects or criteria. This transparency assists decision-makers in comprehending the reasoning behind a particular decision.
- Aggregation operators provide the capability to manage an extensive variety of data and successfully merge and evaluate different criteria, which allows for entire decision-making. This task may prove to be more complex when using soft max-AND and soft min-OR operators.

Based on the study conducted, it becomes apparent that the two techniques presented in this research have notable flexibility and efficacy in comparison to existing problem-solving methodologies.

## Conclusion

Depression, anxiety, drug misuse, and job-related stress are prevalent mental health issues that impact people, their families, coworkers, and the larger society. This research demonstrates how to use the expertise of specialists to determine their preferences for certain mental disorder-related characteristics. While the ambiguity and complexity of crises, together with the unpredictability of the external environment, may place overwhelming pressure on decision-makers. The most recent development of the probabilistic hesitant fuzzy set is the complex probabilistic hesitant fuzzy N-soft set, which aims to handle unexpected situations and psychological behaviour in the surrounding environment together with two-dimensional information in a single set. For this purpose, we introduced the concept of the complex probabilistic hesitant fuzzy N-soft set. Firstly, we discussed its basic or fundamental operations like extended and restricted intersection, extended and restricted union, weak complement, top and bottom weak complement, as well as aggregation operations with their properties. We developed the averaging and geometric aggregation operators to aggregate the information of decision makers effectively. Furthermore, we introduced the decision-making procedure, which provides a more accurate and quicker computing procedure than existing methods. Moreover, we illustrated the examples for the identification of mental disorders, which provides improvements in the treatment of mental health, care quality, and hospitalizations, as well as the potential for enhanced population health. In the last, we compared our proposed model with the existing studies to show its efficacy, superiority, and applicability, as represented in Table 11. We are optimistic that the insights presented here will lay the groundwork for future research, innovation, and decision support systems that help healthcare professionals navigate mental health disorders and improve the well-being and quality of life of those affected. This study shows its research limitations. The article only gives discrete probability information, while continuous probability would better match the real-world situation. Thus, future studies must focus on continuous probability information. Apart from that, we shall extend our approach to analyze the different applications related to emergency supply and the different tools of artificial intelligence, such as optimization or neural networks.

## Data Availability

All data generated or analysed during this study are included in this article.

## Abbreviations

MPGDM: Multi-parameter group decision making
CPHFNS: Complex probabilistic hesitant fuzzy N-soft
CPHFNSS: Complex probabilistic hesitant fuzzy N-soft set
CPHFNSWA: Complex probabilistic hesitant fuzzy N-soft weighted averaging aggregation
CPHFNSWG: Complex probabilistic hesitant fuzzy N-soft weighted geometric aggregation
GCPHFNSWA: Generalized complex probabilistic hesitant fuzzy N-soft weighted averaging aggregation
GCPHFNSWG: Generalized complex probabilistic hesitant fuzzy N-soft weighted geometric aggregation
ACEs: Adverse childhood experiences
GAD: General anxiety disorder
DSM: Statistical manual of mental disorders
